# Insights into the assembly and architecture of a Staufen-mediated mRNA decay (SMD)-competent mRNP

**DOI:** 10.1038/s41467-019-13080-x

**Published:** 2019-11-07

**Authors:** Manjeera Gowravaram, Juliane Schwarz, Sana K. Khilji, Henning Urlaub, Sutapa Chakrabarti

**Affiliations:** 1 Institute of Chemistry and Biochemistry, Freie Universität Berlin, Takustrasse 6, 14195 Berlin, Germany; 2 Bioanalytical Mass Spectrometry Group, Max Planck Institute for Biophysical Chemistry, Am Fassberg 11, 37077 Goettingen, Germany; 3 Bioanalytics Institute for Clinical Chemistry, University Medical Center Goettingen, Robert Koch Strasse 40, 37075 Goettingen, Germany; 4 Present Address: Max Planck Institute of Colloids and Interfaces, Potsdam-Golm Science Park, Am Mühlenberg 1 OT Golm, 14476 Potsdam, Germany

**Keywords:** RNA-binding proteins, RNA decay, Molecular modelling

## Abstract

The mammalian Staufen proteins (Stau1 and Stau2) mediate degradation of mRNA containing complex secondary structures in their 3’-untranslated region (UTR) through a pathway known as Staufen-mediated mRNA decay (SMD). This pathway also involves the RNA helicase UPF1, which is best known for its role in the nonsense-mediated mRNA decay (NMD) pathway. Here we present a biochemical reconstitution of the recruitment and activation of UPF1 in context of the SMD pathway. We demonstrate the involvement of UPF2, a core NMD factor and a known activator of UPF1, in SMD. UPF2 acts as an adaptor between Stau1 and UPF1, stimulates the catalytic activity of UPF1 and plays a central role in the formation of an SMD-competent mRNP. Our study elucidates the molecular mechanisms of SMD and points towards extensive cross-talk between UPF1-mediated mRNA decay pathways in cells.

## Abstract (web summary)

The Staufen proteins recognize secondary structures in 3’-untranslated regions in mRNA transcripts and induce degradation of these mRNAs with the help of the RNA helicase UPF1. Here the authors report that the nonsense-mediated mRNA decay factor UPF2 mediates the interaction between Stau1 and UPF1 in Staufen-mediated mRNA decay.

## Introduction

The fate of a messenger RNA (mRNA) in cells is heavily influenced by the protein factors it associates with throughout its lifetime^1^. Sequence and structural features within the mRNA dictate the proteins bound to it and regulate all aspects of its metabolism, such as processing, transport, translation and decay. In particular, the half-life of mRNA in cells is heavily dependent on specific features, referred to as *cis*-acting elements, usually present in the 3′-untranslated region (3′-UTR) of the mRNA. These *cis*-acting elements are bound by *trans*-acting protein factors, resulting in the assembly of an mRNA–protein complex (mRNP) of defined composition, which in turn act as an adaptor to recruit the mRNA degradation machinery to the transcript. Therefore, assembly of the mRNP is a critical step in mediating decay of the target mRNA through a specific pathway^2^.

The *cis*-acting signals for decay in eukaryotic mRNA vary from simple linear sequences to more complex structural motifs. Extended double-stranded (ds) RNA stretches, both within the coding sequence and the 3′-UTR, are often recognized by the dsRNA-binding protein Staufen^3–5^. While Staufen is known to be a key mRNA transport and localization factor in *Drosophila*^6,7^, binding of the mammalian Staufen paralogs, Stau1 and Stau2, to inter- and intra-molecular RNA duplexes within the 3′-UTR triggers degradation of the target mRNA^8,9^. This degradation process, referred to as Staufen-mediated mRNA decay (SMD), also relies on efficient translation, and recruitment of the RNA helicase up-frameshift 1 (UPF1) to the Staufen-binding sites (SBS) of the target mRNA^10,11^.

**Fig. 1:**
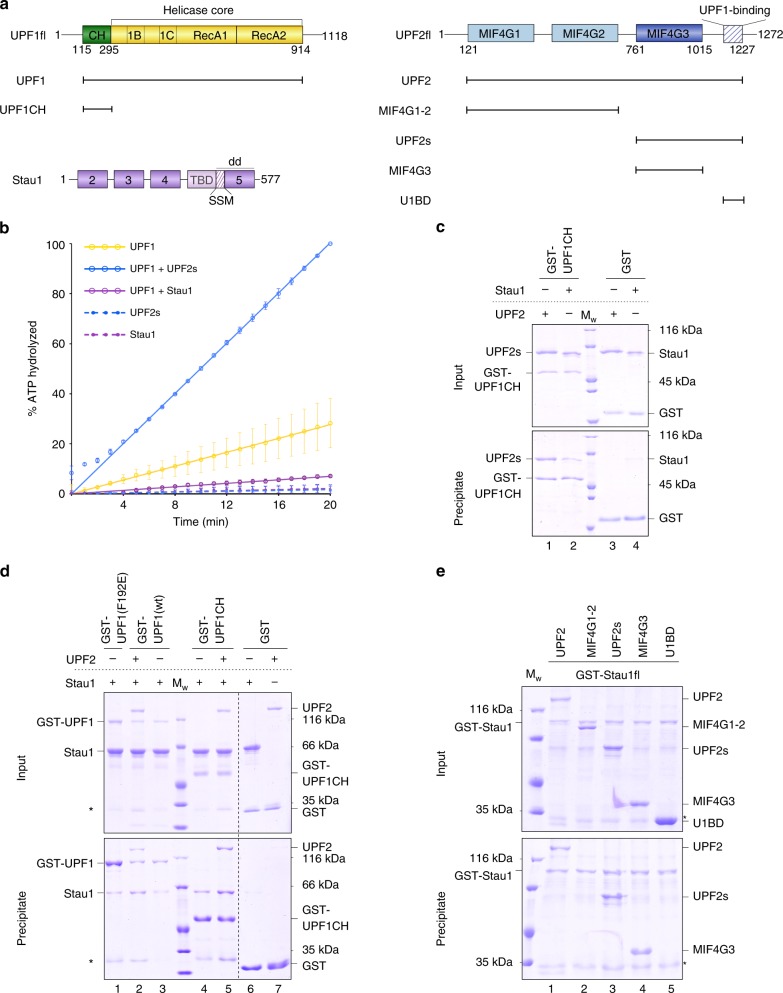
Interactions of Stau1 with UPF1 and UPF2. **a** Schematic representation of the domain arrangements of human UPF1, UPF2, and Stau1. Globular domains are shown as rectangles and flexible linkers and low-complexity regions are denoted as lines. The helicase core of UPF1 consisting of the two RecA domains is colored yellow and the *cis*-inhibitory CH domain is in green. The three MIF4G domains of UPF2 are shown in shades of blue, while the C-terminal UPF1-binding region of UPF2 is shown as a hatched box. The four dsRNA-binding domains (dsRBDs) and the tubulin-binding domain (TBD) of Stau1 are in purple. The Staufen-swapping motif (SSM, hatched box) together with dsRBD5 comprises the Stau1 dimerization module (dd). The constructs of UPF1 and UPF2 used in this study are indicated. **b** RNA-dependent ATPase activity of UPF1 in the presence of UPF2 or Stau1, performed using an enzyme-coupled phosphate detection assay. The data points and their error bars represent the mean values and standard deviation (s.d.) from three independent experiments. **c** GST-pulldown assays of Stau1 and UPF2 with GST-UPF1CH as a bait. GST serves as a negative control. The top and bottom panels indicate input and precipitate in this and all other GST-pulldown experiments. Binding of Stau1 to UPF1CH is ~3-fold weaker than the binding of UPF2 to UPF1CH. **d** GST-pulldown assays of UPF1 and Stau1 in the absence and presence of UPF2. GST-UPF1 and UPF1CH are used as baits while GST serves as a negative control. The presence of UPF2 strengthens the interaction of Stau1 with UPF1 and UPF1CH by ~3-fold. **e** GST-pulldown assays to determine the Stau1-binding region of UPF2. Binding of GST-Stau1 to different UPF2 constructs spanning the entire protein identified the UPF2-MIF4G3 domain as the Stau1-binding site. The corresponding negative controls using GST as bait is shown in Supplementary Fig. 1c. The asterisk (*) indicates contaminants in **d**, **e**. The source data for this figure are provided as source data files

UPF1, a multi-domain protein, is the central component of the nonsense-mediated mRNA decay (NMD) pathway, which detects and degrades mRNA transcripts containing a premature termination codon (PTC)^12^. It consists of an N-terminal cysteine-histidine-rich (CH) domain and a C-terminal helicase core comprising two RecA domains that encompass the nucleotide- and RNA-binding motifs essential for catalysis (Fig. 1a)^13^. The catalytic activity of UPF1 is essential for remodeling nonsense mRNPs to facilitate their degradation and is tightly regulated by intra- and inter-molecular interactions^14–16^. Interaction of the CH domain with the RecA2 domain represses UPF1 activity, whereas binding of the conserved NMD factor UPF2 to the CH domain of UPF1 stimulates its activity. These intra- and inter-molecular interactions are mutually exclusive and are responsible for switching UPF1 from an auto-inhibited state to an activated state by inducing a large conformational change in the helicase upon binding to UPF2 (Supplementary Fig. 1a)^17,18^.

The SMD pathway was also shown to necessitate the helicase activity of UPF1^19^, although it was not clear how the activity is stimulated in the context of SMD. Interestingly, Stau1 can engage in direct protein–protein interactions with the CH domain of UPF1, suggesting that its role in SMD might be analogous to that of UPF2 in the NMD pathway^11^. Furthermore, NMD and SMD were shown to be mutually exclusive pathways, presumably due to the binding of the UPF1CH domain to both UPF2 and Staufen proteins^11^. However, UPF2 and Staufen are considerably different in terms of their primary structure and domain organization. UPF2 consists of three MIF4G (middle portion of eIF4G) domains that typically act as protein–protein interaction platforms, followed by a natively unstructured stretch that binds to UPF1 (referred to as the UPF1-binding domain or U1BD, Fig. 1a)^18,20,21^. Human Stau1 consists of four dsRNA-binding domains (dsRBDs), termed dsRBD2–5 based on their homology with the equivalent domains of *Drosophila* Staufen^22,23^. Of the four dsRBDs, only dsRBD3 and 4 are capable of binding dsRNA^23–26^, while dsRBD5 together with a short sequence located directly N-terminal to it (Staufen-swapping motif, SSM) is responsible for dimerization of Stau1 (Fig. 1a)^27^. Dimerization of Stau1 enhances the efficiency of SMD, possibly by strengthening the interaction of UPF1 with Stau1. Notably, Stau1 lacks a distinct UPF1-binding domain as is found in UPF2 and instead engages UPF1 using its dsRBD4 and Tubulin-binding domains^11^.

In this study, we analyze the interactions between Stau1 and UPF1 in vitro, with an aim to understand the mechanism of recruitment and activation of UPF1 in the SMD pathway. We found that although Stau1 mediates direct interactions with UPF1, this is not sufficient to reconstitute an mRNP. Furthermore, the binding of Stau1 does not stimulate the catalytic activity of UPF1 in vitro. This raises the question of how UPF1 is recruited to an SBS-mRNA and activated in the SMD pathway. Our biochemical reconstitution experiments suggest that the core NMD factor UPF2 plays an integral role in the SMD pathway. We present here a mechanistic study that elucidates how UPF2 mediates the recruitment and activation of UPF1 in the context of SMD and highlights the role of UPF2 in facilitating mRNA decay in this pathway.

## Results

### Stau1 mediates weak interactions with UPF1

Since Stau1 interacts with the *cis*-inhibitory CH domain of UPF1, we hypothesized that, akin to UPF2, Stau1 could stimulate the catalytic activity of UPF1. To this end, we purified a UPF1 construct encompassing its CH domain and helicase core (referred to as UPF1 in the text, Fig. 1a) and tested its RNA-dependent ATPase activity, in the absence and presence of full-length Stau1, using a coupled-enzymatic assay (Fig. 1b). In this study we used the more abundant, shorter splice isoform of UPF1^28^ and the longer isoform of Stau1 (Stau1_63_, referred to as Stau1 throughout). The activity of UPF1 in the presence of a UPF2 construct comprising the MIF4G3 domain and U1BD (UPF2s, Fig. 1a) was measured as a control. As expected, UPF2s robustly stimulated the ATPase activity of UPF1 (Fig. 1b, blue trace). However, full-length Stau1 did not enhance UPF1 activity, even when present in 10-fold molar excess (Fig. 1b, compare yellow and purple traces). To understand the basis of this differential effect of Stau1 and UPF2 on UPF1 activity, we performed glutathione-*S*-transferase (GST)-pulldown assays to compare binding of the two proteins to the UPF1CH domain. A stoichiometric amount of UPF2s was co-precipitated with GST-UPF1CH, whereas the amount of Stau1 detected as being bound to GST-UPF1CH was 3.3-fold lower (Fig. 1c, compare lanes 1 and 2). Furthermore, size-exclusion chromatography (SEC) of a mixture of equi-molar amounts of UPF1 and Stau1 did not yield a stable complex between the two proteins, corroborating the fact that the association of Stau1 with UPF1 is a weak one (Supplementary Fig. 1b). We deduce that the interaction between Stau1 and UPF1 is not strong enough to elicit the large conformational change in UPF1 that is necessary for stimulation of its catalytic activity^17^.

### UPF2 bridges the interaction between Stau1 and UPF1

The inability of Stau1 to form a stable complex with UPF1 also raises the question of how UPF1 is specifically recruited to SBS elements in the 3′-UTR of target mRNAs. UPF1 preferentially binds single-stranded (ss) RNA, suggesting that its recruitment to the structured RNA of the SBS has to be mediated by another protein factor, which either binds the SBS directly or interacts strongly with Stau1. Furthermore, once recruited to the SBS, the helicase activity of UPF1 has to be stimulated to facilitate unwinding of the dsRNA and subsequent degradation. Since UPF2 is the only known activator of UPF1, we proceeded to test if its presence enhances the Stau1–UPF1 interaction. We carried out pulldown assays of GST-tagged UPF1 and UPF1CH constructs with Stau1, in the absence and presence of UPF2 (Fig. 1d). The addition of UPF2 significantly increased the amount of Stau1 that is co-precipitated with UPF1 (3.3- and 2.3-fold increase for UPF1 and UPF1CH, respectively, Fig. 1d, compare lanes 2 and 3 and 4 and 5). We reasoned that UPF2 could augment the binding of Stau1 to UPF1 in two ways: either by acting as an adaptor between Stau1 and UPF1 or by stabilizing an open, active conformation of UPF1 that preferentially binds Stau1. To determine if Stau1 preferentially binds the active form of UPF1, we tested its interaction with a constitutively active mutant, UPF1(F192E). We did not observe stronger binding of Stau1 to activated UPF1, suggesting that the enhancement of Stau1 binding to UPF1 in the presence of UPF2 is not due to selective binding of Stau1 to the open conformation of UPF1 (Fig. 1d, compare lanes 1 and 3).

We next tested if UPF2 functions as an adaptor between UPF1 and Stau1. To this end, we expressed and purified full-length Stau1 with an N-terminal GST tag and performed pulldown assays of GST-Stau1 with UPF2. Surprisingly, UPF2 co-precipitated with GST-Stau1 in stoichiometric amounts, suggesting a robust interaction between the two proteins (Fig. 1e, lanes 1, 3, and 4). Furthermore, co-immunoprecipitation (co-IP) assays of Flag-Stau1 with full-length UPF1 and UPF2 from HEK 293T cells showed that Stau1 interacts with UPF2 in an RNA-independent manner and that binding of Stau1 to UPF1 is enhanced in the presence of UPF2 (Supplementary Fig. 1d). In order to identify the Stau1-binding region of UPF2, we designed a series of UPF2 constructs where each domain was systematically deleted (Fig. 1a) and tested the interaction of these truncated proteins with GST-Stau1. We found that the third MIF4G domain of UPF2 (MIF4G3) was both necessary and sufficient for binding Stau1 (Fig. 1e, lanes 4 and 5). Incidentally, the MIF4G3 domain is also the region of UPF2 that mediates its interaction with the core NMD factor, UPF3, and the eukaryotic release factor eRF3, suggesting that this domain acts an interaction platform in many functional pathways^29–32^. Taken together, our results indicate that UPF2 acts as an adaptor between Stau1 and UPF1 and might play an important role in the recruitment of UPF1 to the SMD pathway.

### The dsRBDs of Stau1 mediate its interaction with UPF2

**Fig. 2:**
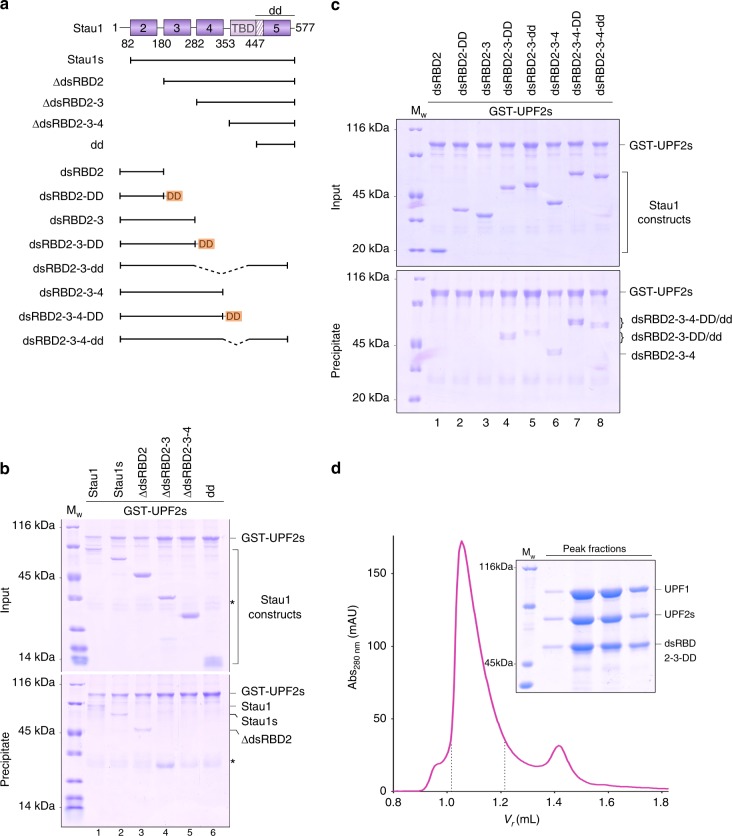
Tandem dsRBDs of Stau1 form a composite binding platform for UPF2. **a** Schematic representation of the Stau1 constructs designed to map the UPF2-binding site. The orange rectangle labeled “DD” represents the dimerization domain of the NF-κB transcription factor p50^33^, connected to Stau1 via a 4-residue linker. The native dimerization module of Stau1, comprising the SSM and dsRBD5 domains, is denoted by “dd”. **b**, **c** GST-pulldown assays of the Stau1 constructs with GST-UPF2s as a bait. Negative controls using GST are shown in Supplementary Fig. 2a, b. The dsRBD3 domain of Stau1 is necessary for binding UPF2, although a strong interaction between UPF2 and Stau1 requires the presence of at least two such binding-competent dsRBDs. Contaminants that co-purified with GST-UPF2 are indicated by asterisks (*). **d** Analytical size-exclusion chromatography (SEC) depicting stable complex formation between UPF1, UPF2s, and a Stau1 construct containing only the UPF2-binding site (dsRBD2-3-DD). The terms Abs and *V*_r_ denote the absorbance and retention volume of the proteins in this and all subsequent chromatograms. The exclusion volume of the column is 0.8 mL. The corresponding SDS-PAGE analysis of the peak fractions (indicated on the chromatogram) is shown on the right. Formation of a stable Stau1-UPF complex is mediated by UPF2. The source data for this figure are provided as a source data file

The human Stau1 protein exists as multiple isoforms in cells. The two major isoforms, referred to as Stau1_63_ and Stau1_55_ in the literature, are identical in their domain structure and organization and differ only in the length of the N-terminal extension preceding the first structured domain, dsRBD2^23^. GST-pulldown assays of GST-UPF2s with the two major Stau1 isoforms (Fig. 2a, Stau1 and Stau1_s_ for the 577- and the 496-residue proteins, respectively) revealed that both isoforms bound UPF2 with equal affinity, therefore indicating that the UPF2-binding site lies within the structured core of Stau1 (Fig. 2b, lanes 1 and 2). To map the UPF2-binding site, we created a series of N-terminal deletion constructs of Stau1, removing successive domains each time (Fig. 2a). Removal of dsRBD2 had no effect on UPF2 binding, whereas deletion of dsRBD3 was sufficient to abolish the interaction between Stau1 and UPF2 (Fig. 2b, compare lane 3 with lanes 4–6). This suggests that the dsRBD3 domain is necessary for mediating the interaction of Stau1 with UPF2.

We attempted to purify the individual dsRBDs of Stau1 in order to precisely locate the UPF2 interaction domain. However, the dsRBD3 and 4 domains were prone to aggregation and could only be purified as fusions with dsRBD2. The resultant constructs, Stau1 dsRBD2-3 and dsRBD2-3-4, were used for GST-pulldown assays. We found that the dsRBD2-3 protein was not sufficient to recapitulate binding of Stau1 to UPF2 (Fig. 2c, lane 3). However, addition of the dsRBD4 domain rescues binding of the protein (Stau1 dsRBD2-3-4) to UPF2 (Fig. 2c, compare lane 6 with 3). Although the dsRBD3 domain of Stau1 is essential for mediating its interaction with UPF2, it appears that the dsRBD4 domain might contribute additional binding sites to assemble a stable interaction platform.

Since the dsRBD3 and 4 domains of Stau1 cooperatively bind RNA^26^, we conjectured that tandem dsRBDs containing UPF2-binding sites are also a requirement for the interaction of Stau1 with UPF2. To test this hypothesis, we engineered constructs of Stau1 dsRBD2-3 fused to a heterologous dimerization domain (that of the nuclear factor-κB (NF-κB) transcription factor p50^33^, indicated by DD) or to its native dimerization module (dd), comprising the SSM and dsRBD5 domains (Fig. 2a). GST-pulldown assays of these Stau1 constructs with GST-UPF2s demonstrated that, in contrast to Stau1 dsRBD2-3, the dimeric proteins were indeed capable of binding UPF2 (Fig. 2c, compare lanes 3, 4, and 5). Fusion of the dimerization modules to the C terminus of Stau1 dsRBD2-3-4 also enhanced its binding to UPF2 (Fig. 2c, compares lanes 6, 7, and 8). Consistent with our observation that deletion of dsRBD2 does not impact the Stau1–UPF2 interaction, we saw no binding upon fusion of the heterologous dimerization domain to Stau1 dsRBD2 alone (Fig. 2c, lane 2). The dimerization domains themselves did not interact with UPF2 (Fig. 2b, lane 6 and Supplementary Fig. 2c). Based on our observations, we propose that at least two interaction domains of Stau1 presented either in *cis*, as in Stau1 dsRBD2-3-4, or in *trans*, as in Stau1 dsRBD2-3-DD/dd, are essential for the formation of a composite interaction platform for UPF2. Incidentally, we found that UPF1 only binds Stau1 in the presence of its native dimerization module or a heterologous dimerization domain. The binding site for UPF1 resides within the Stau1 dsRBD3 and 4 domains, thereby overlapping with its UPF2- and RNA-binding regions (Supplementary Fig. 2d). The pivotal role of UPF2 in bridging the interaction between Stau1 and UPF1 is evident from our SEC analysis where the Stau1 dsRBD2-3-DD protein, which comprises the primary UPF2-binding domain (dsRBD3), but lacks part of the UPF1-binding site (dsRBD4), was still capable of engaging UPF1 in the presence of UPF2 (Fig. 2d).

### Stau1 dimerization impacts recruitment of the UPF proteins

**Fig. 3:**
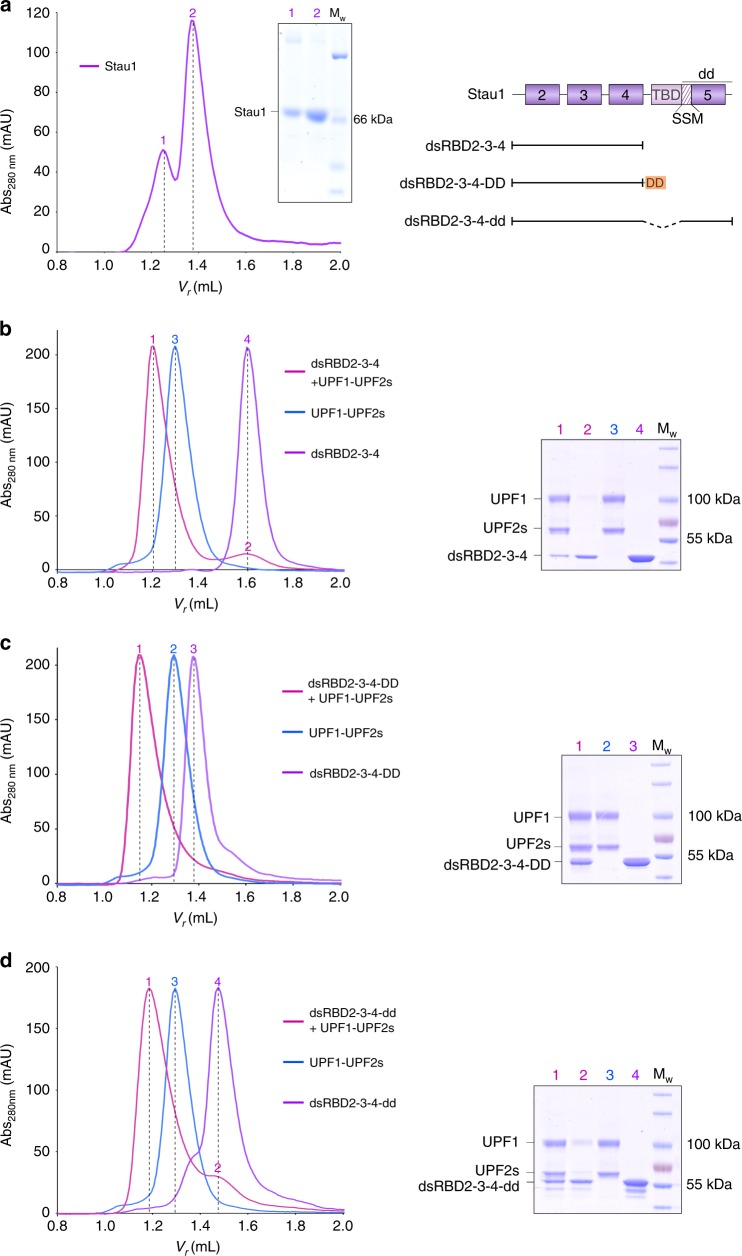
Stau1 dimers promote the assembly of Stau1-UPF ternary complexes. **a** Analytical SEC and corresponding SDS-PAGE analysis illustrating the monomer and dimer populations of full-length Stau1. **b**–**d** Analytical SEC to assess the ability of monomeric and dimeric Stau1 proteins (shown in **a**) to form a stable complex with UPF1 and UPF2s. Left: overlay of chromatograms of the three-component mixture (UPF1-UPF2-Stau1, in pink), the UPF1-UPF2 complex (blue), and the indicated Stau1 protein (purple); right: corresponding SDS-PAGE analyses of the peak fractions of each experiment. The detailed SDS-PAGE analyses of the runs are shown in Supplementary Fig. 3b. Monomeric Stau1 comprising the UPF1 and UPF2 interaction domains does not adequately engage the proteins to form a stoichiometric complex. Fusion of a heterologous or the native dimerization domain to the same Stau1 construct leads to stoichiometric binding of the resultant Stau1 proteins to UPF1 and UPF2. Since the identity of the heterologous dimerization domain (DD) does not affect the behavior of Stau1, a fusion of Stau1 with the dimerization domain of the Hsp70-interacting protein (Hip)^54^ was used for these experiments to achieve clear separation of the proteins by SDS-PAGE. The source data for this figure are provided as a source data file

Full-length Stau1 dimerizes in solution and in cells^34^, although the proportion of dimer in solution is relatively low (Fig. 3a, compare peaks 1 and 2). Previous reports and our multi-angle light scattering (MALS) data suggest that the protein is in rapid equilibrium between the monomeric and dimeric forms (Supplementary Fig. 3a). Dimerization of Stau1 was shown to be essential for mediating SMD, possibly by augmenting its binding to UPF1^27^. In light of our results implicating UPF2 in bridging the Stau1–UPF1 interaction, we set out to investigate the role of Stau1 dimerization in reconstituting a stable UPF1-UPF2-Stau1 ternary complex. First, we tested the ability of the Stau1 dsRBD2-3-4 protein (comprising both UPF1- and UPF2-binding sites, but lacking the dimerization module) to form a stable complex with UPF1 and UPF2. MALS analysis indicated that the Stau1 dsRBD2-3-4 protein is a monomer in solution (Supplementary Fig. 3a). Equi-molar amounts of the three proteins were mixed and analyzed by analytical SEC, together with the UPF1-UPF2 complex and the individual Stau1 protein. Stau1 dsRBD2-3-4 associated in a sub-stoichiometric manner with the UPF proteins and failed to form a stable ternary complex with UPF1 and UPF2 (Fig. 3b, peaks 1 and 2 in the SEC profile and lanes 1 and 2 of the sodium dodecyl sulfate-polyacrylamide gel electrophoresis (SDS-PAGE) analysis). In contrast, the dimeric protein Stau1 dsRBD2-3-4-DD was capable of stable association with UPF1 and UPF2, as indicated by a lower retention volume of the ternary complex compared to that of the UPF1-UPF2 complex (Fig. 3c, compare peaks 1 and 2 of the SEC profile). Furthermore, equal amounts of Stau1 dsRBD2-3-4-DD co-eluted with the UPF proteins, denoting a stoichiometry assembly of the ternary complex (Fig. 3c, SDS-PAGE analysis, lane 1). Fusion of the native dimerization domain (dd) to the Stau1 dsRBD2-3-4 construct resulted in a protein that, like its full-length counterpart, is in rapid equilibrium between a dimer and a monomer (Supplementary Fig. 3a). The Stau1 dsRBD2-3-4-dd protein also engaged UPF1 and UPF2 in a stable interaction, although a significant fraction of the protein was not involved in complex formation (Fig. 3d, peaks 1 and 2 of the SEC profile and lanes 1 and 2 of the corresponding SDS-PAGE analysis). This is in agreement with our observations from GST-pulldown assays where the stronger heterologous dimerization domain had a more significant effect on augmenting UPF2 binding to Stau1 than the weaker native dimerization module (Fig. 2c, compare lanes 4 and 7 with 5 and 8, respectively). These data indicate that dimerization of Stau1 is essential for the stable recruitment of UPF1 and UPF2, and consequently, for all downstream functional effects mediated by the UPF proteins. Domains that are not directly involved in protein–protein interactions within the ternary complex, such as the TBD of Stau1 and the MIF4G1-2 domains of UPF2, did not hinder the assembly of the complex (Supplementary Fig. 3c, d).

### Topology of a UPF1-UPF2-Stau1 ternary complex

**Fig. 4:**
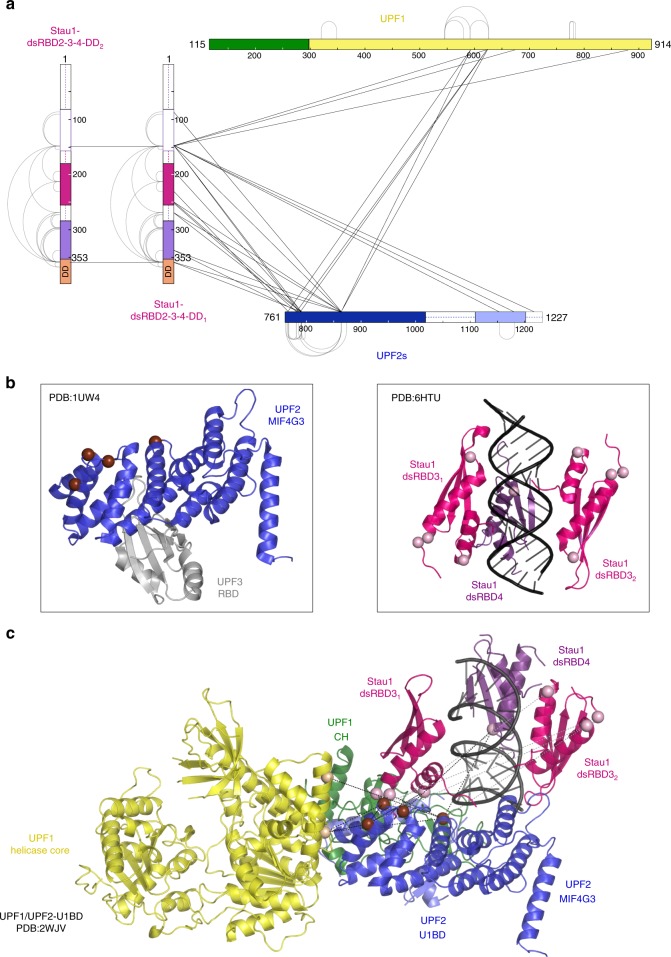
Topology of a UPF1-UPF2-Stau1 complex. **a** Linkage map showing the identified pairwise cross-links between proteins in the Stau1-UPF ternary complex, generated by the program xiNET^53^. The bars for each protein depict the construct used to reconstitute the ternary complex for the cross-linking mass spectrometry analysis. The UPF1 and UPF2 proteins are colored as in Fig. 1. The dsRBD3 and 4 domains of Stau1 and the fused dimerization domain (Hip) are in pink, purple, and orange, respectively. Intra-links within each protein are shown as gray solid lines; inter-links between UPF1 and UPF2 and between a Stau1 monomer to UPF1/UPF2 are shown as black solid lines. Inter-links are only shown with one Stau1 monomer, as the two molecules within the dimer cannot be distinguished in our study. A list of intra-and inter-links for each protein can be obtained from Supplementary Data 1. **b** Previously determined X-ray crystal structures of the UPF2-UPF3 complex (left panel) and Stau1 dsRBD3-4 bound to dsRNA (right panel) with the cross-linked residues highlighted in each case. Residues of UPF2 that cross-link to Stau1 and UPF1 are shown as brown spheres (left panel) while those of Stau1 that cross-link to UPF2 are depicted as pink spheres (right panel). The domains of UPF2 and Stau1 are colored as above, while UPF3 and dsRNA are shown as gray and black cartoons, respectively. **c** Predicted structural model of the UPF1-UPF2-Stau1 ternary complex generated using the Haddock 2.2 Prediction server^35^. Available X-ray crystal structures and distance restraints between pairwise cross-linked residues were used as inputs for prediction. The protein domains, cross-linked residues, and inter-links are depicted as described above

Our biochemical studies suggest that UPF2 acts as an adaptor in the SMD pathway, connecting a Stau1 dimer to the UPF1 helicase. To gain insight into the spatial proximity of the individual proteins within the complex, we performed cross-linking mass spectrometry (MS) using the long-range chemical cross-linker BS3 (bis(sulfosuccinimidyl)suberate). A long spacer arm separates the two functional groups of BS3, allowing it to cross-link lysine residues up to 30 Å apart in three-dimensional space. The analysis was performed on an in vitro reconstituted complex, consisting of UPF1 and the minimal interaction domains of UPF2 and Stau1 (UPF2s and Stau1 dsRBD2-3-4-DD, respectively). In line with our biochemical data, we found extensive cross-links between Stau1 and UPF2 and relatively few cross-links between Stau1 and UPF1 (Fig. 4a). Strikingly, a majority of cross-links between Stau1 and UPF2 mapped to the MIF4G3 domain of UPF2. Cross-links were also found between UPF2-MIF4G3 and the helicase core of UPF1, despite complex formation between the UPF proteins being dictated by interactions between the UPF1CH domain and the U1BD of UPF2. Cross-linking mass spectrometric analysis of the UPF1-UPF2s complex also yielded very few cross-links between the UPF1CH domain and the UPF2 C-terminus (Supplementary Data 1), suggesting that most lysines within the CH domain are not in a favorable position to carry out the cross-linking reaction. The residues of UPF2 that cross-linked to UPF1 (K_781_ and K_790_) and Stau1 (K_785_ and K_863_) mapped to the outer convex surface of the MIF4G3 domain, distinct from its UPF3-binding site (Fig. 4b, left panel, brown spheres). A surprisingly high number of intra-links were found for the Stau1 protein, in comparison to UPF1 and UPF2. We suggest that at least some of these intra-links represent cross-links between two Stau1 monomers, although our analysis did not allow us to distinguish between the individual monomers within the ternary complex.

Using the information on interacting surfaces obtained from our cross-linking mass spectrometric analysis and previously determined X-ray crystal structures, we generated a three-dimensional model of the UPF1-UPF2-Stau1 ternary complex bound to dsRNA using the Haddock 2.2 webserver^35^. From the predicted model, it emerges that the MIF4G3 domain of UPF2 is a central platform around which the UPF1-UPF2-Stau1 complex is assembled (Fig. 4c). Interaction of the UPF1CH domain with the UPF2 C-terminus appears to position the UPF2-MIF4G3 domain in close proximity to the RecA1 domain of UPF1. In this position, the MIF4G3 domain can engage with the dsRBD3 and 4 domains of Stau1, while the N- and C-termini of Stau1 can wrap around the UPF1 RecA domains and the UPF2 C terminus. Interestingly, the residues of dsRBD3 and dsRBD4 that cross-linked to UPF2 map to a solvent-exposed surface of Stau1 that is distal to its RNA-binding interface (Fig. 4b, right panel, pink spheres), implying that binding of UPF2 to Stau1 does not affect its interaction with RNA. Our results suggest that the topology of this ternary complex is compatible with RNA binding, although certain rearrangements might occur upon interaction of the complex with RNA.

### The role of UPF2 in assembling an SMD-competent mRNP

**Fig. 5:**
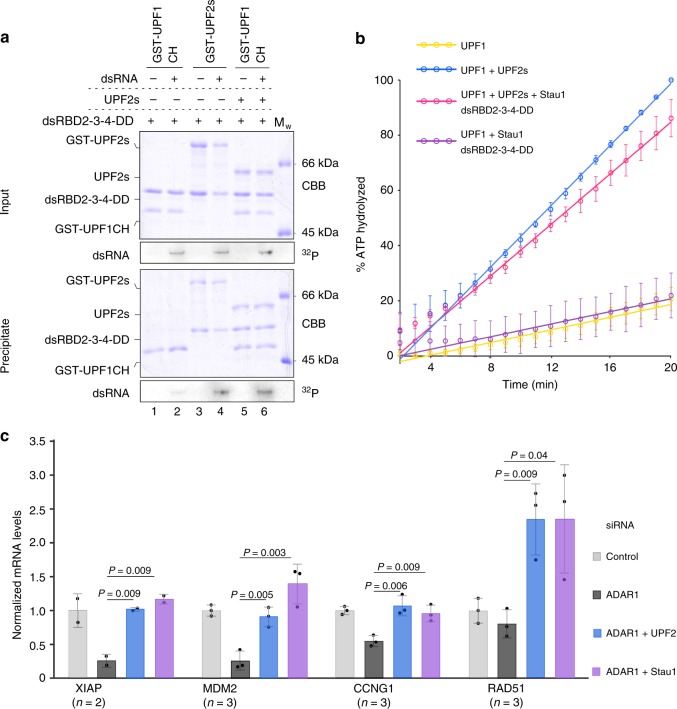
A pivotal role for UPF2 in the SMD pathway. **a** GST-pulldown assays of Stau1 with GST-UPF1CH and GST-UPF2s in the absence and presence of dsRNA. The dsRNA was partially labeled with ^32^P to enable its detection by autoradiography (lower panels of input and precipitate). As before, proteins were detected by staining with Coomassie Brilliant Blue (CBB). The co-precipitation of dsRNA with UPF1 is significantly enhanced in the presence of UPF2 due to strong interactions between UPF2 and Stau1 in the presence of dsRNA. **b** RNA-dependent ATPase of UPF1 in complex with UPF2 and Stau1, performed using an enzyme-coupled phosphate detection assay. The ATPase activity of UPF1 in the presence of either UPF2 or Stau1 served as controls. The data points and their error bars represent the mean values and standard deviation (s.d.) from three independent experiments. **c** Quantitative (q) PCRs to determine levels of known ADAR1/Stau1 targets, *XIAP*, *MDM2*, *CCNG1*, and *RAD51*, upon knockdown of SMD proteins in U2OS cells. Target mRNA levels were normalized to that of the GAPDH transcript in every case. The control siRNA refers to a scrambled sequence that does not specifically target any transcript. Knockdown of UPF2 in combination with ADAR1 leads to increase in the levels of SMD target. The ADAR1/Stau1 knockdown was performed as a positive control. The data were obtained from duplicates of the indicated number of biological replicates, with error bars denoting the standard deviation (s.d.) between the biological replicates. Individual data points are represented as solid circles, while the mean of each data series is represented as a column. The differences in mRNA levels between the ADAR1 knockdown and the ADAR1/UPF2 or the ADAR1/Stau1 knockdown samples is significant, as indicated by the *p* values obtained from unpaired *t* tests. The source data for **a**, **b** are provided as a source data file

Formation of an mRNP consisting of Stau1 and UPF1 is imperative for correctly positioning the helicase with respect to the SBS, such that it can effectively carry out its 5′–3′ unwinding activity. We hypothesize that UPF2 facilitates the recruitment of UPF1 to the Staufen-bound SBS, thereby leading to the assembly of an mRNP, and also enhances UPF1 catalytic activity in this context. To this end, we performed GST-pulldown assays to dissect the interactions between Stau1 and the UPF proteins in the presence of dsRNA, using GST-UPF1CH as bait. The dsRNA was labeled at its 5′ end with ^32^P to enable its detection by autoradiography. Consistent with the weak interaction between Stau1 and UPF1 (Fig. 5a, lanes 1 and 2), the amount of dsRNA co-precipitated with GST-UPF1CH was negligible (Fig. 5a, lower panel). In contrast, the robust interaction between Stau1 and UPF2 led to co-precipitation of significant amounts of dsRNA with GST-UPF2s (Fig. 5a, lanes 3 and 4). The addition of dsRNA did not appear to perturb the interaction between UPF2 and Stau1, validating our observations from cross-linking MS, where UPF2 was found to cross-link to a Stau1 surface distal to its RNA-binding interface (Fig. 4b, right panel). The strong binding between UPF2 and Stau1 is instrumental in recruiting UPF1 to dsRNA-bound Stau1, as indicated by the stoichiometric co-elution of the UPF2 and Stau1 proteins, as well as the dsRNA with GST-UPF1CH (Fig. 5a, lane 6). It should be noted that the CH domain does not interact with RNA in the absence of the UPF1 helicase core. These data suggest that the assembly of an mRNP encompassing Stau1 and UPF1 is driven by the concomitant interaction of UPF2 with SBS-bound Stau1 and UPF1.

We next performed RNA-dependent ATPase assays to assess the catalytic activity of UPF1 in complex with Stau1 and UPF2. As mentioned earlier, Stau1 alone was incapable of stimulating the ATPase activity of UPF1 (Fig. 5b, compare yellow and purple traces, and Supplementary Fig. 4a). However, in the presence of Stau1 and UPF2s, UPF1 exhibited an increase in catalytic activity that is comparable to the increase observed upon addition of UPF2s alone (Fig. 5b, compare yellow, pink, and blue traces, and Supplementary Fig. 4a). Therefore, stimulation of UPF1 ATPase activity in context of the Staufen-mRNP can be attributed to the presence of UPF2 in this complex. Since the binding of UPF2 to UPF1 remained unchanged in the presence of Stau1 (Supplementary Fig. 4b), the activation of UPF1 in the presence of Stau1–UPF2 did not exceed that by UPF2 alone.

Finally, in order to ascertain the contribution of UPF2 in mediating mRNA degradation via SMD, we took advantage of a recent study by Sakurai et al.,^36^ where the RNA-editing enzyme ADAR1 was shown to regulate the levels of specific mRNAs that contain dsRNA structures in their 3′-UTR by binding to them and protecting them from SMD. Down-regulation of ADAR1 in cells leads to an increased binding of Stau1 to the target mRNA and a subsequent reduction in mRNA levels due to SMD. Studies from Yang and co-workers^4,5,36,37^ also showed that ADAR1 binds a subset of Stau1 targets. We performed small interfering RNA (siRNA) knockdowns of ADAR1 alone and in combination with either UPF2 or Stau1 (as a positive control for SMD) in U2OS cells and analyzed the levels of four mRNA transcripts, known to bind both ADAR1 and Stau1^4,5,36,37^, by quantitative reverse transcription PCR (RT-qPCR) (Fig. 5c). The extent of knockdown of each transcript was also verified by RT-qPCR (Supplementary Fig. 4c). Knockdown of ADAR1 led to a significant reduction in the levels of the *XIAP* and *MDM2* transcripts and a more modest reduction in *CCNG1* and *RAD51* levels. However, consistent with published data, a combined knockdown of Stau1 and ADAR1 led to an increase in target mRNA levels due to the absence of SMD in these cells. We found that a knockdown of UPF2 in combination with ADAR1 also led to an increase in mRNA levels, suggesting that SMD was suppressed in the absence of UPF2 (Fig. 5c, compare blue and purple bars). These observations underline the importance of UPF2 in assembling an SMD-competent mRNP in cells and highlight it as a major player of the SMD pathway.

## Discussion

**Fig. 6:**
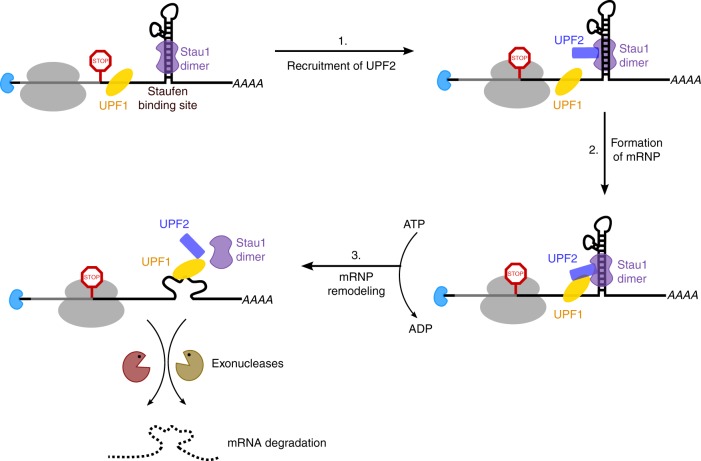
A proposed mechanistic model for SMD. UPF2 plays a key role in recruiting UPF1 to the SBS-mRNA and activating the helicase in the context of SMD

The involvement of RNA helicases in pathways of mRNA decay is a well-established theme in biology^38^. This includes the Ski2 and Mtr4 helicases that are associated with the cytoplasmic and nuclear exosomes, Dhh1 (DDX6/RCK in humans) that mediates mRNA decapping, and a number of helicases such as DHX34, MOV10, and UPF1 that are involved in the NMD pathway^39–42^. In addition to NMD, UPF1 has been implicated in several other mRNA degradation pathways such as SMD, histone mRNA decay, and Regnase-1-mediated decay^10,43,44^. Although the catalytic activity of UPF1 was shown to be essential for mediating mRNA decay in these diverse pathways, other NMD factors that play a role in post-translational modification, activation, and recruitment of UPF1 were not identified in these pathways. In the SMD pathway, these roles were believed to be fulfilled by the Stau1 protein. The prevailing model of SMD suggests that direct interactions between Stau1 and UPF1 recruit the helicase to the SBS of target mRNA and activate it so as to remodel the mRNP in preparation for degradation^8^. Our in vitro biochemical studies using purified proteins have revealed an unexpected role for the core NMD factor UPF2 in the SMD pathway. UPF2 engages in strong interactions with both Stau1 and UPF1, thereby acting as an adaptor between the two hitherto known SMD factors. Using cross-linking MS, we have charted a map for the spatial proximity of three proteins within the ternary complex. Furthermore, we demonstrate the formation of an mRNP comprising UPF1, UPF2, Stau1, and dsRNA and show that UPF1 can be robustly activated by UPF2 in the presence of Stau1, which is a prerequisite for its function in SMD. Indeed, knockdown of UPF2 appears to repress SMD in cells, emphasizing its involvement in this pathway (Fig. 6). Interestingly, a previous study that ascertained a role for UPF1 in SMD did not identify UPF2 as an adaptor between the two proteins or as an essential factor for this pathway^10^.

NMD and SMD are competitive pathways in cells^11^. This was presumed to be due to binding of UPF2 and Stau1 to the same region (CH domain) of UPF1. However, our studies indicate that despite the overlap in the binding site, interactions of Stau1 and UPF2 to UPF1 are not mutually exclusive with each other. On the contrary, binding of UPF2 to UPF1 augments the Stau1–UPF1 interaction. Therefore, the competition between the two pathways cannot be attributed to competition in binding of UPF2 and Stau1 to UPF1. Furthermore, the binding of UPF1 to Stau1 is significantly weaker than to UPF2, suggesting that Stau1 is unlikely to displace UPF2 from UPF1. Interestingly, UPF2 binds to Stau1 through its MIF4G3 domain, which is also the binding site of the core NMD factor UPF3^29^. Although our cross-linking mass spectrometric data suggest that the surface of the UPF2-MIF4G3 domain that contacts Stau1 is distinct from its UPF3-binding site, it is possible that steric hindrances prevent UPF2 from simultaneously interacting with UPF3 and Stau1. We anticipate that the observed competition between the NMD and SMD pathways is not because of competition in binding to UPF1, but rather because of partial overlap in the UPF2-binding sites.

It is still unclear how UPF2 is specifically recruited to the target mRNA prior to degradation. Both Stau1 and UPF1 are RNA-binding proteins and bind ds- and ss-RNA throughout the length of the transcript^5,45^. On the other hand, UPF2 does not bind RNA and must be specifically recruited through its interactions with the RNA-binding factors. However, it is important that this recruitment occurs at a precise time-point in the pathway, as early activation of UPF1 would lead to remodeling of the mRNP and premature mRNA decay. The interactions mediated by UPF2 in the context of NMD provide some clues as to how the timely recruitment of UPF2 to SMD might be achieved. Stalling of the ribosome on a stop codon leads to recruitment of the eukaryotic release factors 1 and 3 (eRF1/3)^46^. Studies by Lopez-Perrote et al.^30^ showed that UPF2 binds eRF3 using its MIF4G3 domain and that this interaction is mutually exclusive with its binding to UPF3. In light of these data, it is tempting to speculate that, in NMD, UPF2 is delivered or handed over from the translation termination complex to UPF3, which is bound to the exon-junction complex present downstream of the premature termination codon. Since SMD also relies upon the presence of an upstream termination codon, a similar hand-over mechanism can be envisaged for recruiting UPF2 to the Stau1-bound mRNA in this pathway.

In contrast to the above-mentioned work, recent studies reported the direct interaction of UPF3 with the eRF1/3 complex in the absence of UPF2, leading to delayed translation termination in NMD^47^. Given that human Stau1 was shown to associate with ribosomes, it is possible that the molecular determinant linking SMD to translation termination is Stau1 and that UPF2 is recruited at a later stage in the pathway^25^.

Previous studies from Gleghorn et al.^27^ suggest that dimerization of Stau1 is essential for its function in SMD, presumably by boosting its interaction with UPF1. Our studies demonstrate the need for a Stau1 dimer in stably interacting with UPF1 and UPF2, rationalizing the observed importance of dimerization in SMD. However, Stau1 forms only weak dimers in solution due to domain-swapping interactions between its dsRBD5 domain and the Staufen-swapping motif (SSM), collectively referred to as the dimerization module. Very recently, Lazzaretti et al.^26^ showed that the Stau1 dimerizes upon binding to dsRNA, independent of its dimerization module, implying that the intrinsically weak dimer might be strengthened upon binding of Stau1 to its target RNA^26^. Dimerization upon RNA binding has been observed for other dsRNA-binding proteins such as NF90 and the adenosine deaminase ADAR2^48,49^. However, unlike NF90, which has a separate hetero-dimerization domain, Stau1 uses the same domains (dsRBD3 and 4) to interact with RNA as well as the UPF proteins. Although certain atypical dsRBDs have been shown to interact with both proteins and dsRNA, the binding events were mutually exclusive^50^. To the best of our knowledge, Stau1 represents the first example of a dsRBD that is capable of concomitant binding to dsRNA and a protein factor. Together with the aforementioned studies on dimerization and RNA binding, our studies also highlight the scope of interactions mediated by dsRBDs and their versatility in cellular functions.

In summary, we have identified the NMD factor UPF2 as the pivotal component that is essential for recruiting the RNA helicase UPF1 to a Staufen-mRNP, activating it within this complex and mediating SMD (Fig. 6). Our findings highlight the mechanistic similarities between NMD and SMD and point to an extensive cross-talk between these, and possibly other UPF1-dependent pathways in cells. Nevertheless, whether UPF2 is involved in every such pathway and how it might be recruited to these pathways remains a topic for further studies.

## Methods

### Protein expression and purification

All human UPF1, UPF2, and Stau1 constructs used in this study were expressed as 6×-His, His-GST, or His-Thioredoxin (Trx) fusions (cleavable with TEV or 3C protease, please refer to Supplementary Table 1) in *Escherichia coli* BL21 (DE3) STAR pRARE cells at 18 °C for at least 15 h. Cells expressing recombinant proteins were lysed using buffer A (20 mM Tris-HCl, pH 7.5, 10 mM imidazole, 1 mM MgCl_2_, 1 µM ZnCl_2_, and 10% glycerol) supplemented either with 500 mM NaCl (for UPF1 and UPF2) or 1 M NaCl (for Stau1), protease inhibitors, and DNase I. The proteins were enriched from the crude lysate by Ni^2+^-affinity chromatography and eluted from the Ni^2+^-NTA resin using buffer A supplemented with an additional 240 mM imidazole and 150 mM NaCl (for UPF1 and UPF2) or 1 M NaCl (for Stau1). The affinity tags on UPF1 and UPF2 were not removed in any case except for His-GST-UPF2 (126–1227), where the N-terminal His-GST tag was removed by cleavage with TEV protease. The His-Trx tags of the Stau1 constructs were cleaved by 3C protease during overnight dialysis at 4 °C against dialysis buffer (20 mM Tris-HCl, pH 7.5, 400 mM NaCl, 10% glycerol).

All proteins were subjected to a further purification step using a HiTrap Heparin Sepharose HP column (GE Healthcare). SEC (Superdex 200 column, GE Healthcare) was performed in buffer B (20 mM HEPES, pH 7.5, 150 mM NaCl, 2 mM dithiothreitol (DTT), 5% glycerol, 1 mM MgCl_2_, and 1 µM ZnCl_2_) as a final step of purification.

To purify a UPF1-UPF2 complex for analytical SEC assays, equi-molar amounts of purified UPF1 and UPF2 were mixed and incubated at 4 °C for 16 h, following which the mixture was injected on a Superdex 200 column in buffer B.

The UPF1-UPF2-Stau1 complex was formed by mixing the purified UPF1-UPF2 complex (described above) with Stau1 in a 1:1 molar ratio (considering Stau1 as a dimer). The protein mixture was incubated for 16 h at 4 °C in buffer B supplemented with 50 mM NaCl (final salt concentration of 200 mM NaCl). The resultant ternary complex was isolated by SEC (Superdex 200 column) in buffer B.

### GST-pulldown assays

GST-pulldown assays were done by mixing equal amounts of bait and prey proteins and diluting the protein mixture to 40 µL in GST-pulldown buffer (20 mM HEPES, pH 7.5, 150 mM NaCl, 10% glycerol, and 0.1% NP-40). The reaction mixture was incubated at 4 °C for 16 h, following which 12 µL of a 50% slurry of Glutathione Sepharose resin (GE Healthcare) was added. The mixture was further supplemented with 200 µL of GST-pulldown buffer and incubated at 4 °C for 1 h. The beads were extensively washed with GST-pulldown buffer. Bound proteins were eluted in GST-pulldown buffer supplemented with 20 mM glutathione and analyzed by SDS-PAGE and Coomassie staining. The intensities of bands in the pulldown gels and the resultant fold change were quantified and calculated using the program ImageJ (https://imagej.nih.gov/ij/). For pulldowns containing dsRNA, we used a chemically synthesized 28-mer, which was shown by Ramos et al.^24^ to fold into a 12-bp stem with an intervening tetraloop between the two arms of the stem (IBA solutions, 5′-GGACAGCUGUCC(GUAA)GGACAGCUGUCC-3′, tetraloop sequence is within parentheses). To check the presence of dsRNA in the input and precipitate samples, the dsRNA was 5′ end labeled with [γ-^32^P]ATP using T4 polynucleotide kinase. The reactions were set up in duplicate, and a 1.2-fold molar excess of 28-mer dsRNA (of which approximately one-third consisted of the labeled dsRNA) was added to the protein mixture after the overnight incubation. The reaction mixture was further incubated at 25 °C for 2 h. Bound proteins and RNA were captured and eluted as described above. One set of eluates were resolved by 7.5% SDS-PAGE and stained with Coomassie Brilliant Blue to visualize the proteins, while the second set of eluates were resolved on a 20% Urea-PAGE and analyzed by autoradiography to detect the labeled dsRNA. The unformatted gels for all GST-pulldown assays, as well as all other experiments, are provided as a Source Data file.

### Analytical SEC

Seven hundred picomoles of the UPF1-UPF2 complex (described above) were mixed with 700 pmol of the dimeric Stau1 proteins (dsRBD2-3-DD, dsRBD2-3-4-DD, and dsRBD2-3-4-dd) and with 1400 pmol of Stau1 dsRBD2-3-4 to a final volume of 40 µL in buffer A (supplemented with 50 mM NaCl) and incubated at 4 °C for 16 h. The peak fractions were analyzed by SDS-PAGE, followed by staining with Coomassie Brilliant Blue.

### ATPase assay

The ATPase activity of UPF1 in the presence of its binding partners was determined by quantifying the amount of inorganic phosphate released upon ATP hydrolysis using a coupled colorimetric assay (EnzCheck Phosphate Kit, Thermo Fisher Scientific)^28^. The protein mixtures (see below) were pre-incubated with 2 μg poly-U RNA, 40 nmol MESG (2-amino-6-mercapto-7-methylpurine ribonucleoside), and 0.5 U purine-nucleoside phosphorylase in reaction buffer (50 mM MES, pH 6.5, 50 mM potassium acetate, 5 mM magnesium acetate, 2 mM DTT) at 30 °C for 20 min. The reaction was initiated by the addition of ATP to a final concentration of 1 mM. Inorganic phosphate released from ATP hydrolysis reacted with MESG to produce 2-amino-6-mercapto-7-methylpurine, which was detected by measuring absorbance at 360 nm on an Infinite M1000 Pro (Tecan). The reaction was monitored over a 20 min period at 60-s intervals. The amount of UPF1 in every experiment was kept constant (6 pmol), and UPF2 was added in 1.25× excess of UPF1, wherever indicated. In Fig. 1, the full-length Stau1 protein was added in 10× excess of UPF1, while the experiments in Fig. 5 contained 7.5 pmol (1.25× of UPF1) of the dimeric Stau1 proteins. The end point of the ATPase reaction (20-min time-point) of the UPF1-UPF2 mixture was set to 100% and all other data points were normalized to this value. The raw data for all ATPase assays are provided as a Source Data file.

### siRNA knockdown and RT-qPCR analysis

U2OS cells were cultured in Dulbecco’s modified Eagle’s medium (DMEM) supplemented with 10% fetal bovine serum (FBS) (Bio&SELL), 100 U/mL of penicillin, and 100 μg/mL of streptomycin (Thermo Fisher Scientific). The siRNAs used for knockdown of ADAR1, Stau1, and UPF2 were procured from Thermo Fisher Scientific (see below). Twenty picomoles of each siRNA (alone or in combinations, as indicated) were transfected into 0.15 × 10^6^ U2OS cells using Lipofectamine 2000 (Thermo Fisher Scientific), as per the manufacturer’s instructions. Total RNA was extracted from U2OS cells 72 h post transfection using RNATri (Bio&SELL). Two hundred nanograms of total RNA was used for gene-specific complementary DNA synthesis by the MLV-reverse transcriptase (Qiagen). Quantitative PCRs (qPCRs) were performed using the PowerUp SYBR Green Master Mix (Thermo Fisher Scientific) on a Stratagene Mx3005P instrument. The data represent the mean of duplicates of three biological replicates, while the error bars denote the standard deviations. Unpaired *t* tests were performed to calculate the *p* values, with the significance threshold set at 0.05.

The qPCR primers used in this study are described in Supplementary Table 2.

The following siRNAs used in this study were obtained from Thermo Fisher Scientific: ADAR1 (catalog # S1008), UPF2 (catalog # S24948), and Stau1 (catalog # S13547). The control siRNA had the following sequence: UUUGUAAUCGUCGAUA CCC-dTdT. The U2OS and HEK 293T (used for co-IP assays, see below) cell lines were a gift from Florian Heyd and were routinely tested for mycoplasma contamination in the course of the study. Cell lines showed the expected morphology and were not authenticated by other methods.

### Cross-linking MS of the UPF-Stau1 complex

Ten micrograms of aliquots of the protein complexes (UPF1-UPF2-Stau1 and UPF1-UPF2) were initially cross-linked with 0.2, 0.5, 1, and 2 mM BS3 (Thermo Scientific) to optimize the minimum concentration of BS3 necessary to achieve efficient cross-linking. The final cross-linking reaction was carried out with 0.2 mM BS3 for 30 min at room temperature and quenched with 50 mM Tris. Samples were separated by SDS-PAGE (NuPAGE 4–12% gradient gel, Invitrogen). The cross-linked complex was cut out of the gel and separated into three pieces. Excised gel pieces were then subjected to in-gel tryptic digest^51^. Samples were reduced with 10 mM dithiotreitol and alkylated with 55 mM iodacetamide and subsequently digested with trypsin (sequencing grade, Promega) at 37 °C for 18 h. Extracted peptides were dried in a SpeedVac Concentrator and dissolved in loading buffer composed of 4% acetonitrile and 0.05% trifluoroacetic acid. Samples were subjected to liquid chromatography MS on a QExactive HF-X (Thermo Scientific). Peptides were loaded onto a Dionex UltiMate 3000 UHPLC + focused system (Thermo Scientific) equipped with an analytical column (75 µm × 300 mm, ReproSil-Pur 120 C18-AQ, 1.9 µm, Dr. Maisch GmbH, packed in house). Separation by reverse-phase chromatography was done on a 60-min multi-step gradient with a flow rate of 0.3–0.4 µL/min. MS1 spectra were recorded in profile mode with a resolution of 120k, maximal injection time was set to 50 ms, and automatic gain control (AGC) target to 1e^6^ to acquire a full MS scan between 380 and 1580*m*/*z*. The top 30 abundant precursor ions (charge state 3–8) were triggered for high-energy collision-induced dissociation fragmentation (30% normalized collision energy). MS2 spectra were recorded in profile mode with a resolution of 30k; maximal injection time was set to 128 ms, AGC target to 2e^5^, isolation window to 1.4*m*/*z*, and dynamic exclusion was set to 30 s. Raw files were analyzed via pLink2.3.5 to identify cross-linked peptides, with standard settings changed as follows: peptide mass: 600–10,000; precursor tolerance: 10 p.p.m.; fixed modification: carbamidomethyl [C] and variable modification oxidation: [M]^52^. False discovery rate was set to 1% and results were filtered by excluding cross-links supported by only one cross-linked peptide spectrum match. The interaction network for the ternary complex was illustrated via xiNET^53^. The analysis was carried out with one sample each for the ternary and the UPF1-UPF2 binary complex (in one and three technical replicates for the ternary and binary complexes, respectively).

The structural model of the UPF1-UPF2-Stau1 ternary complex was generated using the Haddock 2.2 Prediction server^35^. Available X-ray crystal structures (UPF1-UPF2, PDB ID 2WJV; UPF2-UPF3, PDB ID 1UW4; Stau1-dsRNA, PDB ID 6HTU) and distance restraints between pairwise cross-linked residues (Supplementary Data 1) were used as inputs for prediction. The modeling was performed in a sequential manner where cross-links between UPF1 and the UPF2-MIF4G3 were modeled first and the resultant output was used to map cross-links between Stau1 and UPF2-MIF4G3. The outputs with the lowest Haddock score were considered for analysis. The final model was generated by superposing the UPF2-MIF4G3 domains from both runs.

### SEC-MALS assay

Approximately 350 µg of each Stau1 construct was injected onto a Superdex 200 10/300 column (GE Healthcare) on an Agilent HPLC system coupled to miniDAWN TREOS (Wyatt Technology, Germany) and RefractoMax520 (ERC) detectors. Proteins were eluted using a running buffer containing 20 mM HEPES, pH 7.5, 150 mM NaCl, and 0.02% NaN_3_. The instrument was calibrated using bovine serum albumin as a reference. Data recording and analysis was performed using the ASTRA 6.1.4.25 software (Wyatt Technology, Germany). The experimentally determined and theoretical molecular weights are mentioned in Supplementary Fig. 3a.

### Co-IP assays

HEK 293T cells were cultured in DMEM supplemented with 10% FBS (Bio&SELL) and Pen/Strep (Invitrogen). A total of 0.5 × 10^6^ cells/ml were seeded in a 6-well plate format, and full-length constructs of Flag-Stau1, HA-UPF1, and HA-UPF2 were co-transfected using polyethyleneimine “Max” (Polysciences Inc., *M*_w_ 40,000). Transfected cells were incubated at 37 °C and harvested 48 h later. Cells were lysed using 200 µL of RIPA buffer (10 mM Tris-HCl, pH 7.5, 100 mM NaCl, 2 mM EDTA, 1% NP-40, supplemented with protease inhibitors) and RNase A (final concentration of 60 µg/mL). Fifteen microliters of anti-Flag M2 resin (Merck, catalog # A2220) was added to each cell lysate sample and incubated at 4 °C for 1.5 h. Unbound proteins were washed with RIPA buffer and Flag-tagged baits and the associated proteins were eluted in non-reducing SDS sample buffer by incubating at 30 °C for 3 h. The eluted samples were resolved by 10% SDS-PAGE and then transferred to a PVDF membrane for analysis by western blotting with mouse anti-HA (1:4000; Covance, catalog # MMS-101R), mouse anti-Flag (1:4000; Merck, catalog # F1804) antibodies wherever indicated. The secondary antibody horse anti-mouse horse radish peroxidase (1:4000; Cell Signaling Technologies, catalog # 7076S) was used to enable detection of tagged proteins by chemiluminescence.

### Reporting summary

Further information on research design is available in the Nature Research Reporting Summary linked to this article.

## Supplementary information

Supplementary Information

Peer Review

Reporting Summary

Description of Additional Supplementary Files

Supplementary Dataset 1

Source Data

## Data Availability

A reporting summary for this article is available as a Supplementary Information file. All data generated or analyzed during this study are included in this published article and its Supplementary Information files, or available from the corresponding author upon reasonable request. The source data underlying Figs. 1, 2, 3, and 5a, b and Supplementary Figs. 1, 2, 3, and 4a, b are provided as Source Data files. The raw data corresponding to Fig. 4 is provided in Supplementary Data 1.
