# Supplementary material for: Insights into the assembly and architecture of a Staufen-mediated mRNA decay (SMD)-competent mRNP
Source: Nat Commun. 2019 Nov 7;10:5054. doi: 10.1038/s41467-019-13080-x (PMC6838198; doi:10.1038/s41467-019-13080-x)
Supplement: Supplementary file 1 — Supplementary Information [file 41467_2019_13080_MOESM1_ESM.pdf]

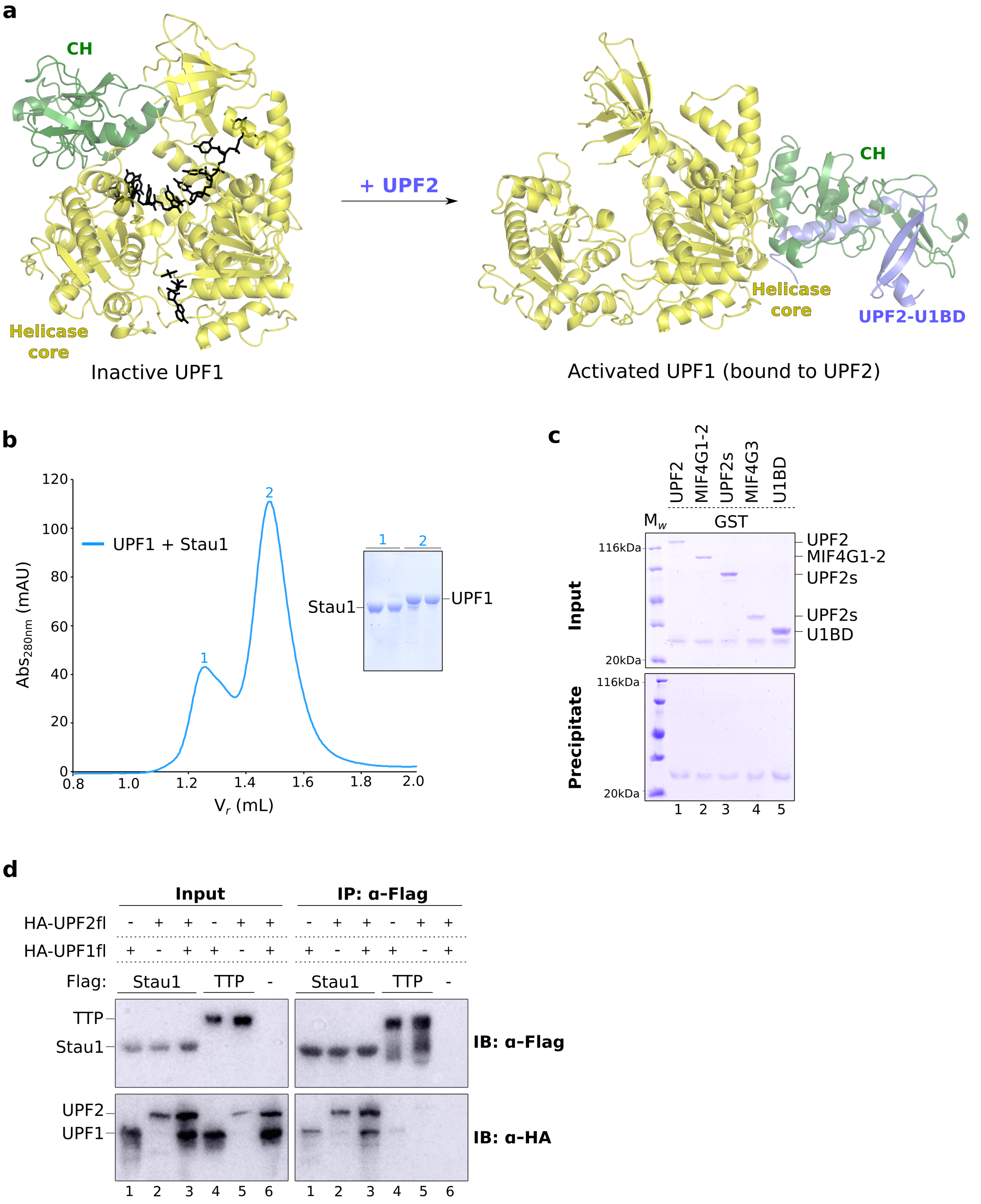

Supplementary Fig. 1 (related to Fig. 1 of the main text) (a) Previously determined X-ray crystal structures show the changes involved in the activation of UPF1 by UPF2. Interaction of the CH domain with the RecA2 domain maintains the helicase in an inactive conformation (PDB ID: 2XZL[1]) whereas binding of UPF2 to the CH domain of UPF1 switches it to an active conformation (PDB ID: 2WJV[2]). (b) Analytical SEC to assess complex formation between UPF1 and Stau1 when mixed in equi-molar amounts. The corresponding peak fractions analyzed in SDS-PAGE show that UPF1

and Stau1 elute in two separate peaks, denoting absence of a stable complex. (c) GST-pulldown assay of UPF2 constructs with GST as a bait for negative control (refer to Fig. 1d in the main text). (d) Co-immunoprecipitation (IP) assays of Flag-Stau1 full length and HA-UPF1 and UPF2 (full-length proteins). The constructs were co-transfected in HEK 293T cells and cell lysates were subjected to IP using the anti-Flag (M2) resin. 5% of the total cell lysate of every sample was used as the input. Inputs and precipitates were analyzed by SDS-PAGE and immunoblotting using the indicated antibodies. Flag-GFP-TTP and anti-Flag M2 resin were used as negative controls. The left and right panels indicate input and precipitate.

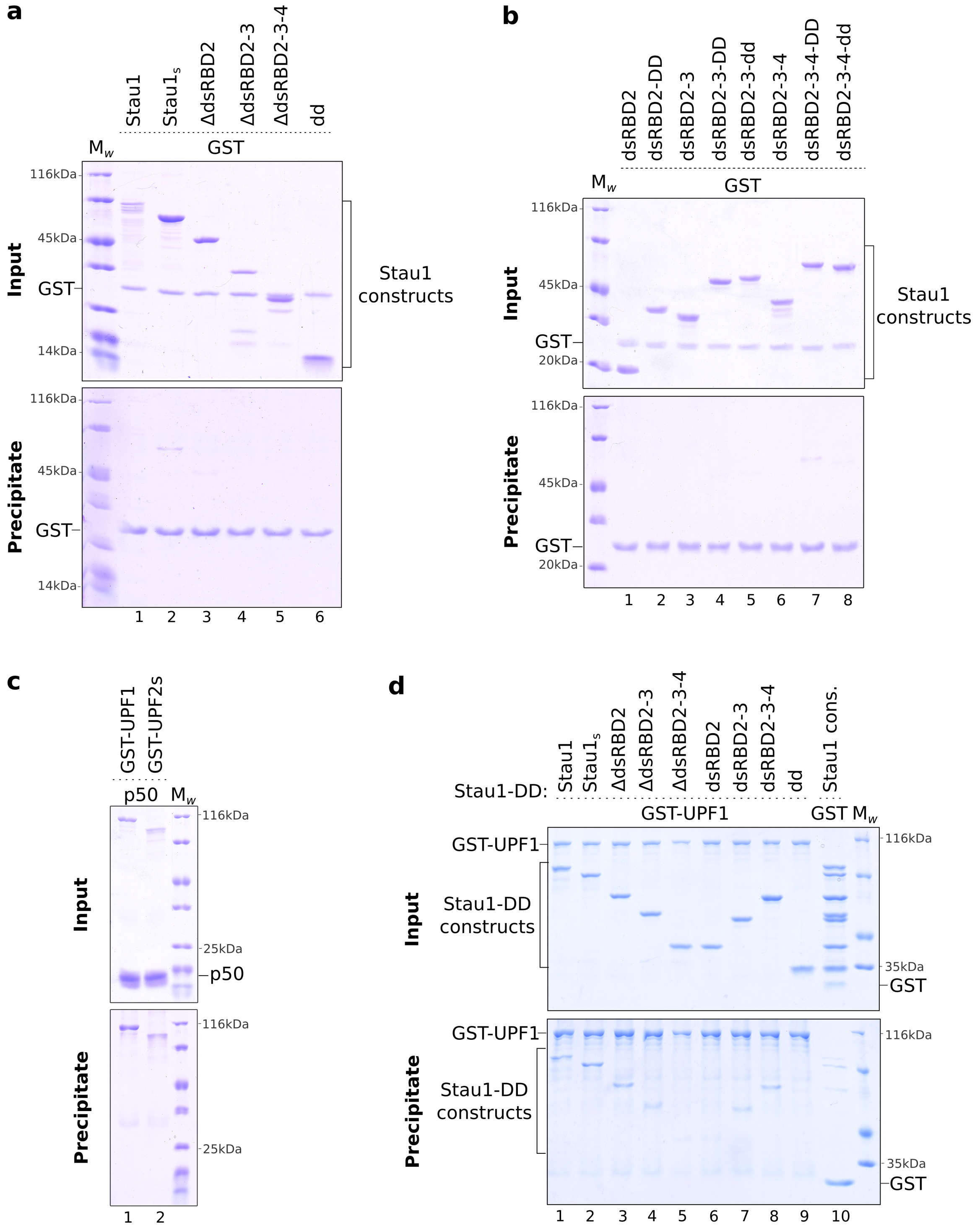

Supplementary Fig. 2 (related to Fig. 2 of the main text) (a) and (b) Negative controls using GST as bait for the GST-pulldown assays of the Stau1 constructs (refer to Fig. 2b and 2c in the main text). The top and bottom panels indicate input and precipitate in all pulldown experiments. (c) GST-pulldown assay showing the absence of interaction between GST-UPF1 and GST-UPF2s with the heterologous dimerization domain, p50 (DD). (d) GST-pulldown assays of the Stau1 constructs with GST-UPF1 as bait shows

the involvement of dsRBD3 and 4 in binding to Stau1. GST was used as negative control, against all the Stau1 constructs.

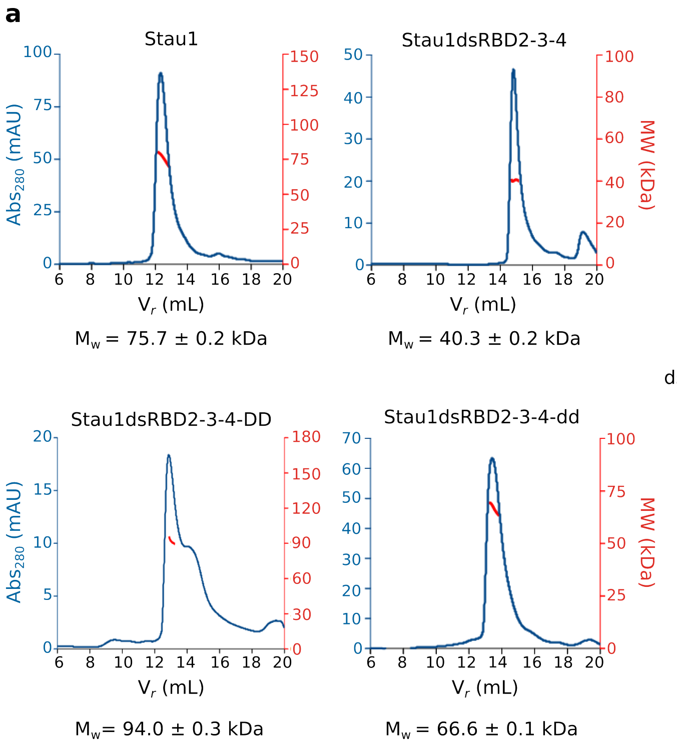

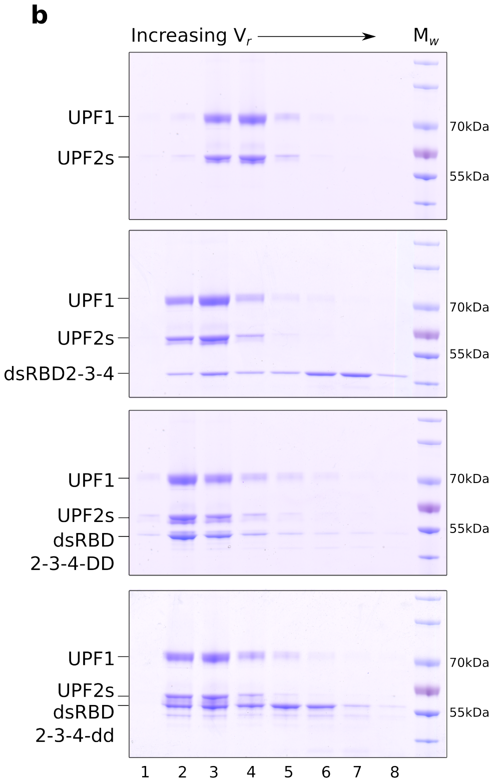

Sample	Theoretical M_w (kDa)	Experimental M_w (kDa)
Stau1	63.7 (monomer)<br>127.4 (dimer)	75.7
Stau1dsRBD2-3-4	39.3 (monomer)<br>78.6 (dimer)	40.3
Stau1dsRBD2-3-4-DD	44.6 (monomer)<br>89.2 (dimer)	94.0
Stau1dsRBD2-3-4-dd	54.4 (monomer)<br>108.8 (dimer)	66.6

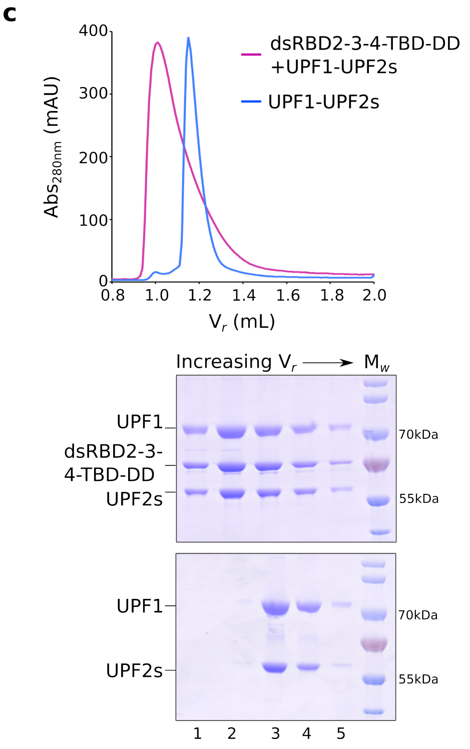

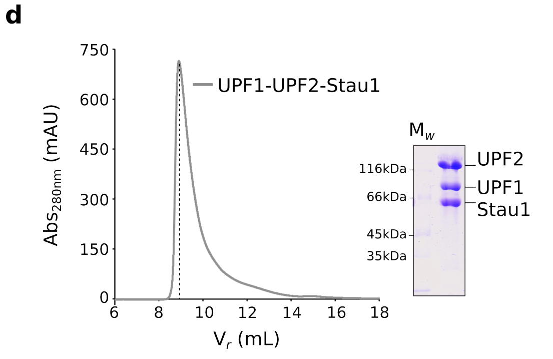

Supplementary Fig. 3 (related to Fig. 3 of the main text) (a) SEC-MALS was used to determine the molar mass (kDa) across the peaks for purified Stau1 constructs. Experimentally obtained values are indicated below each panel. The table denotes the

expected molar mass for monomeric and dimeric forms of each Stau1 construct (calculated using the Expasy Protparam tool) and the experimental molar mass obtained from SEC-MALS analysis. (b) Coomassie-stained 3-8% NuPAGE Tris-acetate gels used to analyze the fractions of the SEC runs of UPF1-UPF2 complex with different Stau1 constructs. Lane numbers indicate the elution fraction for each SEC run in ascending order of the retention volume (increasing V_r). (c) Analytical SEC depicting the ability of a Stau1 construct containing the tubulin-binding domain (dsRBD2-3-4-TBD-DD) to form a stable complex with UPF1 and UPF2. The proteins were mixed in equi-molar amounts, as described in the main text. The corresponding SDS-PAGE analysis of the peak fractions is shown at the bottom. Lane numbers indicate the elution fractions as described above. (d) Semi-analytical SEC denoting the reconstitution of a ternary complex with near-full length constructs of UPF1 (residues 115-914), UPF2 (residues 126-1227) and full-length Stau1 (refer to Fig. 1a in the main text for domain boundaries of UPF1 and UPF2). The Stau1 protein was added in 1.5-fold molar excess to UPF1 and UPF2. An SDS-PAGE analysis of the peak fraction (indicated on the chromatogram) is shown on the right.

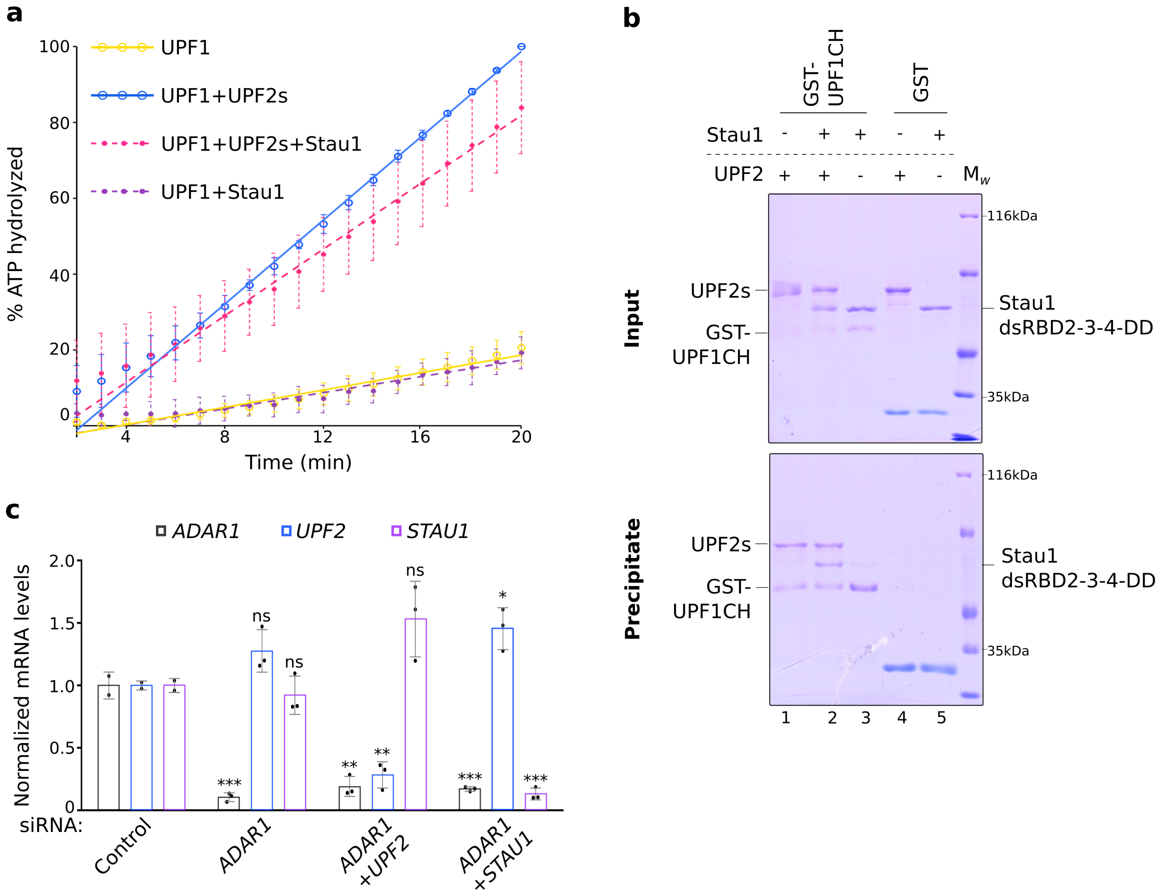

Supplementary Fig. 4 (related to Fig. 5 of the main text) (a) RNA-dependent ATPase activity of UPF1 in the presence of UPF2s or full-length Stau1, performed using an enzyme-coupled phosphate detection assay. The data points and their error bars represent the mean values and standard deviation from three independent experiments. (b) GST-pulldown assays to compare the binding of UPF2 to GST-UPF1CH in the absence and presence of Stau1. GST serves as a negative control. The binding of UPF2 to UPF1 is unaffected by the presence of Stau1. (c) RT-qPCR assays to verify levels of ADAR1, UPF2 and Stau1 mRNA in each of the siRNA knockdowns performed. The mRNA levels were normalized to that of the GAPDH transcript in every case. The control siRNA refers to a scrambled sequence that does not specifically target any transcript. Solid circles, columns and errors bars represent the individual data points, the mean of each data series and the standard deviation (s.d.) within each data series, respectively. Significant differences were identified by unpaired t-tests (* $P < 0.05$, ** $P < 0.01$, *** $P < 0.001$, ns. not significant). Only those mRNA levels targeted by the siRNA were significantly reduced while others remained unaffected.

Supplementary Table 1. Plasmids used in this study

E. coli expression (Vector backbone for all plasmids: pET)

	Gene	Construct Boundaries	Comments	Expression tags
1	UPF1	115-914	CH-helicase domains *	N-term. His_6
2	UPF1	115-914	CH-helicase domains *	N-term. His_6-GST
3	UPF1	115-914, F192E	Hyperactive mutant of UPF1	N-term. His_6-GST
4	UPF1	115-272	CH domain	N-term. His_6-GST
5	UPF2	126-1227	MIF4G1-2-3 + UPF1 binding site *	N-term. His_6-GST
6	UPF2	761-1227	MIF4G3 + UPF1 binding site *	C-term. His_6
7	UPF2	761-1227	MIF4G3 + UPF1 binding site	N-term. His_6-GST
8	UPF2	121-757	MIF4G1-2	N-term. His_6
9	UPF2	761-1054	MIF4G3	N-term. His_6
10	UPF2	1015-1227	UPF1 binding site	C-term. His_6
11	Stau1	1-577	full-length **	N-term. His_6-GST
12	Stau1	1-577	Full-length	N-term. His_6-Trx
13	Stau1	82-577	Short isoform	
14	Stau1	180-577	ΔdsRBD2	
15	Stau1	282-577	ΔdsRBD2-3	
16	Stau1	362-577	ΔdsRBD2-3-4	
17	Stau1	447-577	TBD-SSM-dsRBD5	
18	Stau1	1-182	dsRBD2	
19	Stau1	1-282	dsRBD2-3	
20	Stau1	1-353	dsRBD2-3-4	
21	p50	245-350	Dimerization domain (DD) of mouse p50	
22	Stau1-p50	1-577-DD	C-terminal fusion with p50DD Stau1 domains as described above	
23	Stau1-p50	82-577-DD		
24	Stau1-p50	180-577-DD		
25	Stau1-p50	282-577-DD		
26	Stau1-p50	362-577-DD		
27	Stau1-p50	447-577-DD		
28	Stau1-p50	1-182-DD		
29	Stau1-p50	1-282-DD		
30	Stau1-p50	1-353-DD		
31	Stau1-p50	1-441-DD		
32	Stau1	1-282-GSGS-441-577	Residues 283-440 replaced by GSGS	
33	Stau1	1-353-GSGS-441-577	Residues 354-440 replaced by GSGS	
34	Stau1-Hip	1-353-DD	C-terminal fusion with HipDD	

Mammalian cell expression

	Vector Backbone	Gene	Construct Boundaries	Comments	Expression tags
1	pFlag-CMV2	UPF1	1-1118	Full-length, Flag tag replaced by HA	N-term. HA
2	pEFα-HA	UPF2	1-1272	Full-length	N-term. HA, C-term. His
3	pEFα-λN-Flag	Stau1	1-577	Full-length, native sequence of human Stau1 [#]	N-term. Flag
4	pEGFP-N3	TTP	1-326	Full-length	N-term His-Flag, C-term GFP

* Plasmids were a gift from Elena Conti

** This and all other Stau1 plasmids for *E. coli* expression were derived from a pET28a-Stau1 plasmid that was codon-optimised for expression in *E.coli* (gift from Fulvia Bono)

[#] Gift from Fulvia Bono

Supplementary Table 2. Primers used in this study

Primers used for cloning plasmids in Supplementary Table 1

Primer Name	Primer description	Primer sequence
**oSC32**	Stau1_fwd_(1)	caggggcccgactcgatgtcacaagtccaagtcc
**oSC33**	Stau1_rev_(577)	cagaccgccaccgactgcttattaacaacggccgcaca
**oSC62**	Stau1(577)_fwd_p50fusion	gtgtgcggccgttgtgcaggagcatccaacctgaaaatc
**oSC63**	Stau1(577)_rev_p50fusion	gattttcaggttggatgctcctgcacaacggccgcacac
**oSC72**	p50_rev_(350)	cagaccgccaccgactgcttattcagggtagtagagaaag
**oSC87**	p50_fwd_(245)	ccaggggcccgactcgatggcatccaacctgaaaatcg
**oSC119**	Stau1_rev_(182)	cagaccgccaccgactgcttagttcaggttttcttcttcg
**oSC122**	Stau1(182)_fwd_p50fusion	cgaagaagaaaacctgaacggaagtgcaggcgcatccaacctgaaaatcg
**oSC123**	Stau1(182)_rev_p50fusion	cgattttcaggttggatgcgcctgcacttccgttcaggttttcttcttcg
**oSC137**	Stau1_fwd_(82)	ccaggggcccgactcgatgaaactgggcaaaaagc
**oSC142**	Stau1_fwd_(362)	ccaggggcccgactcgatgacgaagccggcactgaaaag
**oSC143**	Stau1_fwd_(447)	ccaggggcccgactcgatggcaaaggctaccgtgacg
**oSC144**	Stau1_rev_(441)	cagaccgccaccgactgcttaacgggtgaagtctttcg
**oSC145**	Stau1_rev_(353)	cagaccgccaccgactgcttagcccaggatttccagc
**oSC146**	Stau1_rev_(282)	cagaccgccaccgactgcttaaccatattccggtgagg
**oSC147**	GA_p50DD_rth	gcaggagcatccaacctgaaaatcg
**oSC148**	Stau1(441)_rth_3'	tgcacgggtgaagtctttcgtgtg
**oSC149**	Stau1(353)_rth_3'	gcccaggatttccagcatattttcagc
**oSC150**	Stau1(282)_rth_3'	accatattccggtgaggtctgcgg
**oSC156**	Stau1(282)_fwd_p50fusion	gcagacctcaccggaatatggtagtgcaggcgcatccaacctgaaaatcg
**oSC157**	Stau1(282)_rev_p50fusion	cgattttcaggttggatgcgcctgcactaccatattccggtgaggtctgc
**oSC158**	Stau1_fwd_(180)	ccaggggcccgactcgatgaacctgaacaagagcg
**oSC159**	Stau1_fwd_(282)	ccaggggcccgactcgatgggtcaaggcattaacccg
**oSC169**	Stau1(577)_Hipfusion_fwd	cgtgtgcggccgttgtgcaggaatggatccccgcaaagtg
**oSC170**	Stau1(577)_ Hipfusion_rev	cactttgcggggatccattcctgcacaacggccgcacacg
**oSC181**	GA_HipDD_rth	gcaggaatggatccccgcaaagtgagc
**oSC224**	Stau1_281_GSAGSA_rev	gctgcagatccagcactaccaccatattccggtgaggtctg
**oSC225**	Stau1_353_GSAGSA_rev	gctgcagatccagcactaccgcccaggatttccagcatattttc
**ES205**	UPF1_(115)_fwd	ccagggagcagcctcgatgacgaaggacctccccatac
**ES145**	UPF1_(914)_rev	gcaaagcaccggcctcgttagctgaactgcatgag
**oSC30**	UPF1_272_rev	gcaaagcaccggcctcgttagttttccttccacagctc
**oSK7**	UPF1_Flag-HA_CMV2_rev	gcgtaatctggaacatcgtatgggtacatggtagatcaattc
**oSK8**	UPF1_Flag-HA_CMV2_fwd	ccatacgatgttccagattacgctcttgcggccgcgaattcatcg
**oSC39**	hUPF2(761)_fwd	ccagggagcagcctcgatggaaaaaaccgtgaaaaag
**oSC44**	hUPF2(1227)_rev	gcaaagcaccggcctcgttaatcttcttgttcttgc
**oSC60**	hUPF2(1054)_rev	gcaaagcaccggcctcgttaatttacttcactttcatttcc
**VN07F**	hUPF2(121)_fwd	ccaggggcccgactcgatgaaagaaaaagaagaatccattcagc
**VN10R**	hUPF2(757)_rev	cagaccgccaccgactgcttatgggttgcagtagtaatatgc
**oSC276**	UPF2(1015)NLIC_3C	Ccaggggcccgactcgatgaatgacaaagactc
**oSC48**	UPF2(1)N_Mfe1	atgccaattgcatgccagctgagcg
**oSC49**	UPF2(1272)CHis_Not1	aattgcggccgctcaatggtgatggtgatggtgtgcacgtctcctcccac

qPCR primers

Primer description	Primer sequence
GAPDH_Fwd	CTTCGCTCTCTGCTCCTCCTGTTCG
GAPDH_Rev	ACCAGGCGCCC AATACGACCAAAT
UPF2_Fwd	TTGGTACGGGCACTCTTCAT
UPF2_Rev	CAT CAGACATGCAGGGATGC
Stau1_Fwd	GAGTACACGCTCCTCACAGA
Stau1_Rev	CCTTCTGCAGTGTGGTTTCC
ADAR1_Fwd	CTAACAGCTTCCAGCCCTCC
ADAR1_Rev	AGCGATTCCCTGTTCCCAAG
XIAP_Fwd	TACCGTGCGGTGCTTTAGTT
XIAP_Rev	TTTGTAGACTGCGTGGCACT
MDM2_Fwd	ATGAAAGCCTGGCTCTGTGT
MDM2_Rev	AAGATCCGGATTCGATGGCG
CCNG1_Fwd	AACTGGATTATTACAGCACC
CCNG1_Rev	CTTATCTCGTGATGTTTTGAGG
RAD51_Fwd	CAGATTGTATCTGAGGAAAGG
RAD51_Rev	ATGATTCAGTCTTTGGCATC

Supplementary references

1. Chakrabarti, S. et al. Molecular mechanisms for the RNA-dependent ATPase activity of Upf1 and its regulation by Upf2. *Mol Cell* **41**, 693-703 (2011).
2. Clerici, M. et al. Unusual bipartite mode of interaction between the nonsense-mediated decay factors, UPF1 and UPF2. *EMBO J* **28**, 2293-306 (2009).
