# Supplementary material for: Insights into the assembly and architecture of a Staufen-mediated mRNA decay (SMD)-competent mRNP
Source: Nat Commun. 2019 Nov 7;10:5054. doi: 10.1038/s41467-019-13080-x (PMC6838198; doi:10.1038/s41467-019-13080-x)
Supplement: Supplementary file 3 — Reporting Summary [file 41467_2019_13080_MOESM3_ESM.pdf]

natureresearch

Corresponding author(s): Sutapa Chakrabarti

Last updated by author(s): Sep 22, 2019

Reporting Summary

Nature Research wishes to improve the reproducibility of the work that we publish. This form provides structure for consistency and transparency in reporting. For further information on Nature Research policies, see Authors & Referees and the Editorial Policy Checklist .

Statistics

For all statistical analyses, confirm that the following items are present in the figure legend, table legend, main text, or Methods section.

n/a	Confirmed	
☐	☒	The exact sample size (n) for each experimental group/condition, given as a discrete number and unit of measurement
☐	☒	A statement on whether measurements were taken from distinct samples or whether the same sample was measured repeatedly
☒	☐	The statistical test(s) used AND whether they are one- or two-sided<br>*Only common tests should be described solely by name; describe more complex techniques in the Methods section.*
☒	☐	A description of all covariates tested
☒	☐	A description of any assumptions or corrections, such as tests of normality and adjustment for multiple comparisons
☒	☐	A full description of the statistical parameters including central tendency (e.g. means) or other basic estimates (e.g. regression coefficient) AND variation (e.g. standard deviation) or associated estimates of uncertainty (e.g. confidence intervals)
☐	☒	For null hypothesis testing, the test statistic (e.g. F, t, r) with confidence intervals, effect sizes, degrees of freedom and P value noted<br>*Give P values as exact values whenever suitable.*
☒	☐	For Bayesian analysis, information on the choice of priors and Markov chain Monte Carlo settings
☒	☐	For hierarchical and complex designs, identification of the appropriate level for tests and full reporting of outcomes
☒	☐	Estimates of effect sizes (e.g. Cohen's d, Pearson's r), indicating how they were calculated

Our web collection on statistics for biologists contains articles on many of the points above.

Software and code

Policy information about availability of computer code

Data collection	No special software was used for data collection
Data analysis	Size-exclusion data (Figure 2 and Figure 3) were analyzed using the Unicorn software (version 7.0.2) from GE Healthcare. Cross-links obtained from the cross-linking mass-spectrometric analysis were visualized by the open source program xiNET (Figure 4). The three dimensional model of the ternary protein complex bound to RNA was generated using the web server Haddock (Haddock 2.2 Prediction server).

For manuscripts utilizing custom algorithms or software that are central to the research but not yet described in published literature, software must be made available to editors/reviewers. We strongly encourage code deposition in a community repository (e.g. GitHub). See the Nature Research guidelines for submitting code & software for further information.

Data

Policy information about availability of data

All manuscripts must include a data availability statement. This statement should provide the following information, where applicable:

- Accession codes, unique identifiers, or web links for publicly available datasets
- A list of figures that have associated raw data
- A description of any restrictions on data availability

All data generated or analysed during this study are included in this published article and its supplementary information files, or available from the corresponding author upon reasonable request. The source data underlying Figs. 1, 2, 3, and 5a-b and Supplementary Figs. 1, 2, 3 and 4a-b are provided as Source Data files. The raw data corresponding to Fig. 4 is provided in Supplementary Data 1.

Field-specific reporting

Please select the one below that is the best fit for your research. If you are not sure, read the appropriate sections before making your selection.

- [x] Life sciences
- [ ] Behavioural & social sciences
- [ ] Ecological, evolutionary & environmental sciences

For a reference copy of the document with all sections, see nature.com/documents/nr-reporting-summary-flat.pdf

Life sciences study design

All studies must disclose on these points even when the disclosure is negative.

Sample size	The in vitro biochemical assays such as ATPase assays (reported in Figure 1 and Figure 5) were performed 3 times. The reported data is the mean of the 3 independent experiments. The siRNA knockdown and qPCR experiment reported in Figure 5 is the mean of the number of biological replicates indicated in the figure (n=2 for XIAP, n=3 for all other targets) , of which each replicate was measured twice for technical accuracy.
Data exclusions	One entire set of siRNA knockdowns and qPCR assays were discarded because of discrepancy between the two measurements that were performed for technical accuracy (see above) in more than one sample. We believe that this discrepancy is due to an error in sample preparation. For the target XIAP, an additional set of samples were accidentally lost (handling error).
Replication	All biochemical experiments were performed at least 3 times to ensure reproducibility of the observations.
Randomization	This is not relevant to our study as we did not perform experiments or statistical analysis on large sample sets.
Blinding	This is not relevant to our study as we did not perform experiments or statistical analysis on large sample sets.

Reporting for specific materials, systems and methods

We require information from authors about some types of materials, experimental systems and methods used in many studies. Here, indicate whether each material, system or method listed is relevant to your study. If you are not sure if a list item applies to your research, read the appropriate section before selecting a response.

Materials & experimental systems

n/a	Involved in the study	
[ ]	[x]	Antibodies
[ ]	[x]	Eukaryotic cell lines
[x]	[ ]	Palaeontology
[x]	[ ]	Animals and other organisms
[x]	[ ]	Human research participants
[x]	[ ]	Clinical data

Methods

n/a	Involved in the study	
[x]	[ ]	ChIP-seq
[x]	[ ]	Flow cytometry
[x]	[ ]	MRI-based neuroimaging

Antibodies

Antibodies used	Anti-Flag (M2) antibody, Merck (catalog # F1804); Anti-HA antibody, Covance (MMS-101R)
Validation	These are commercially available antibodies that have been extensively used to detect the Flag and HA epitope tags.

Eukaryotic cell lines

Policy information about cell lines

Cell line source(s)	U2OS cell line: Homo sapiens bone osteosarcoma cell line, HEK 293T cell line: Human embryonic kidney cell line expressing a mutant version of the SV40 large T antigen.
Authentication	Cell lines were obtained from the lab of Florian Heyd. They showed the expected morphology and were not authenticated by other methods.
Mycoplasma contamination	The cell lines tested negative for mycoplasma contamination.
Commonly misidentified lines (See ICLAC register)	We did not use any such cell lines in our study.
