# Supplementary material for: Insights into the assembly and architecture of a Staufen-mediated mRNA decay (SMD)-competent mRNP
Source: Nat Commun. 2019 Nov 7;10:5054. doi: 10.1038/s41467-019-13080-x (PMC6838198; doi:10.1038/s41467-019-13080-x)
Supplement: Supplementary file 4 — Description of Additional Supplementary Files [file 41467_2019_13080_MOESM4_ESM.docx]

**Description of Additional Supplementary Files**

File Name: Supplementary Data 1
Description: Details of cross-linking mass-spectrometry and the analysis to determine pair-wise cross-linked residues of the UPF1-UPF2sStau1dsRBD2-3-4-DD and the UPF1-UPF2s complexes. The table consists of the output files generated by pLink, the summary of cross-linked positions, the pLink outputs filtered by excluding crosslinks supported by one cross-linked peptide match, the filtered summary of cross-linked positions and the fasta sequences used for pLink search.
