# Supplementary material for: Insights into the assembly and architecture of a Staufen-mediated mRNA decay (SMD)-competent mRNP
Source: Nat Commun. 2019 Nov 7;10:5054. doi: 10.1038/s41467-019-13080-x (PMC6838198; doi:10.1038/s41467-019-13080-x)
Supplement: Supplementary file 6 — Source Data [file 41467_2019_13080_MOESM6_ESM.zip › 204899_2_supp_4099594_pyff0v.pdf]

1c

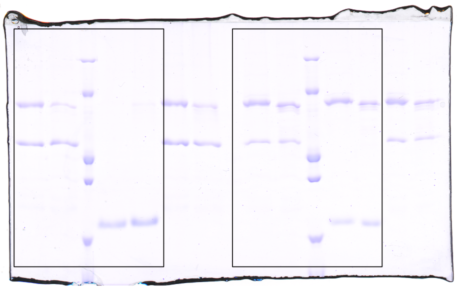

1d

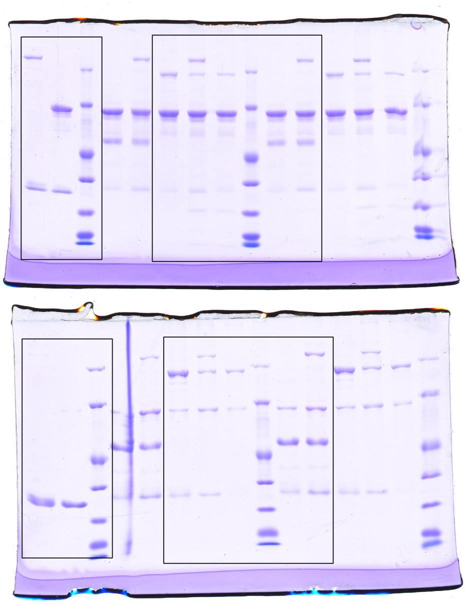

1e

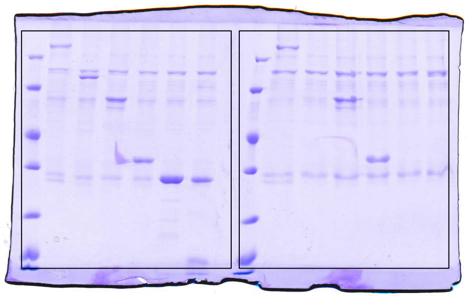

Suppl.Fig. 1c

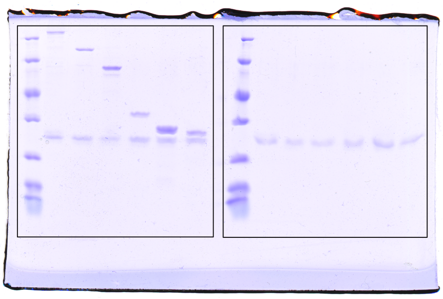

Suppl.Fig. 1b

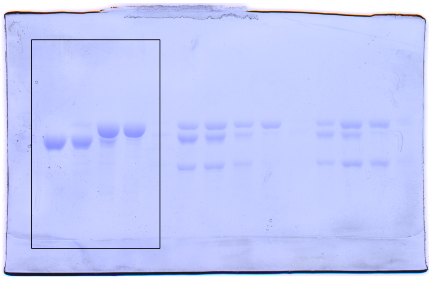

Suppl.Fig. 1d

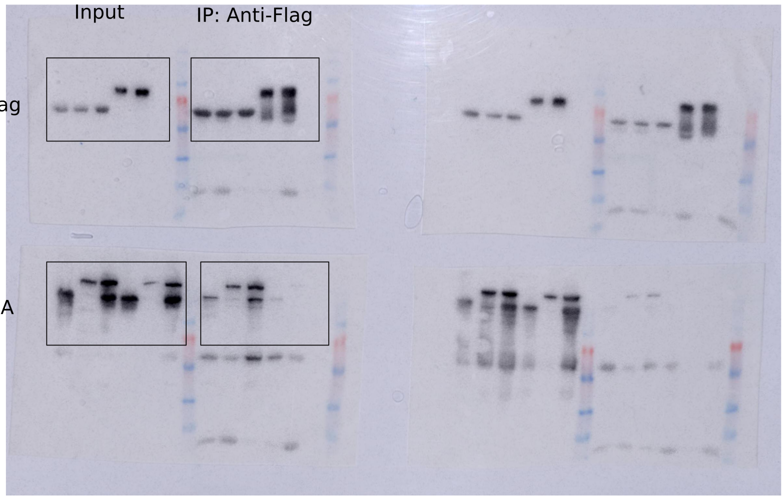

Raw gels for Fig.1 and Supplementary Fig. 1. The regions of the gels/blots highlighted by black boxes in this and all other figures indicate the part of the gels used to generate the final figures. Figure numbers in the source data file refer to the corresponding figures in the main text or supplementary information.

2b

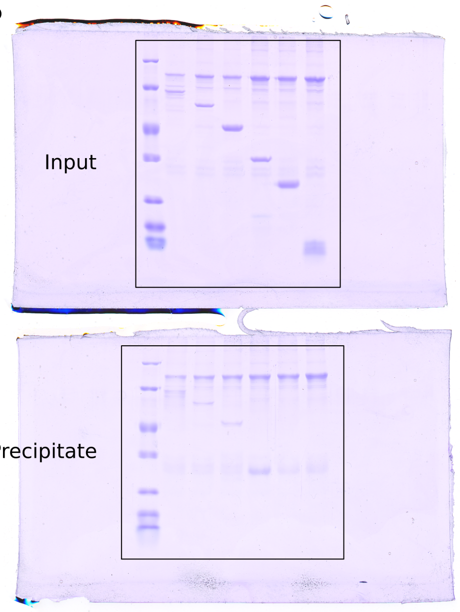

2c

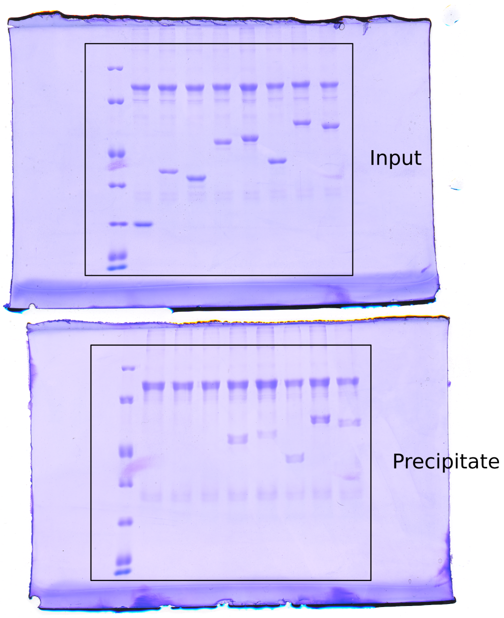

2d

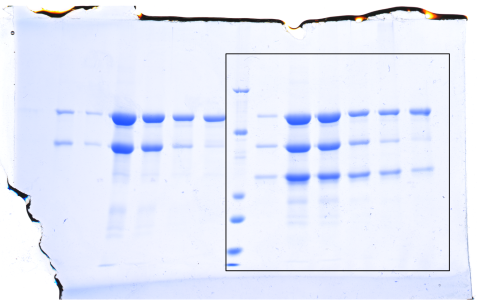

Suppl.Fig. 2a

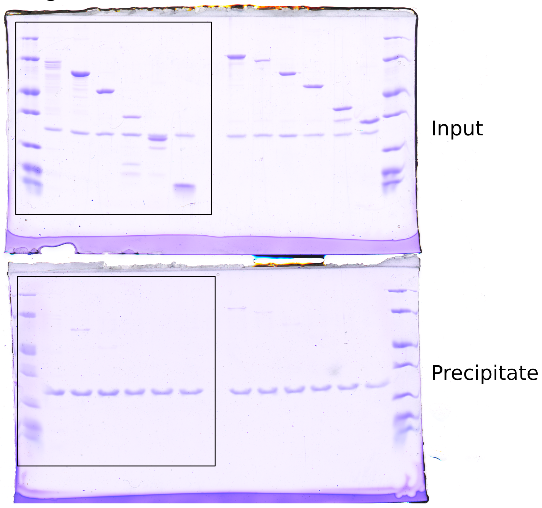

Suppl.Fig. 2b-2c

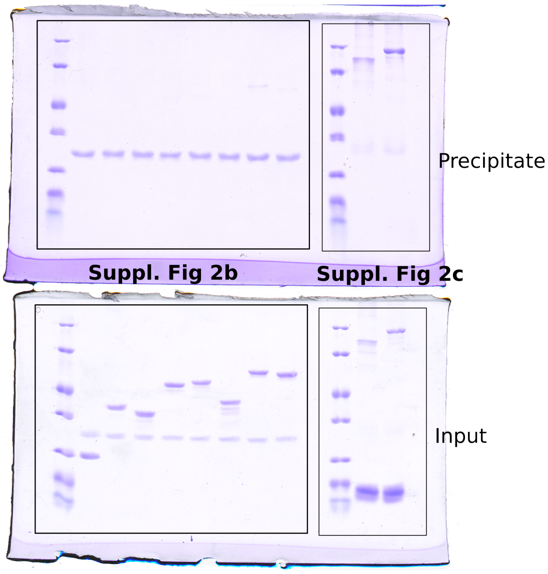

Suppl.Fig. 2d

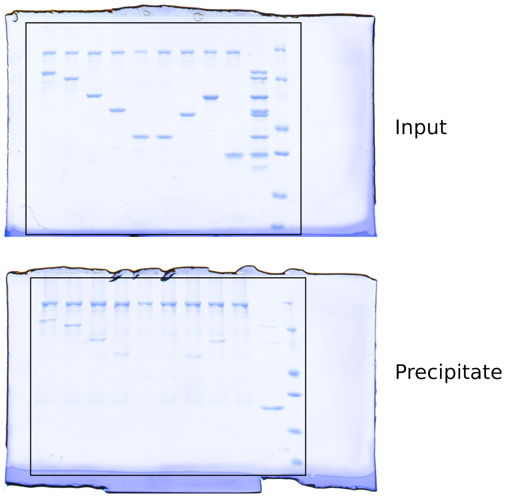

Raw gels for Fig.2 and Supplementary Fig. 2

3a

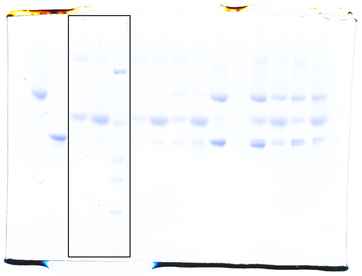

3b-3d

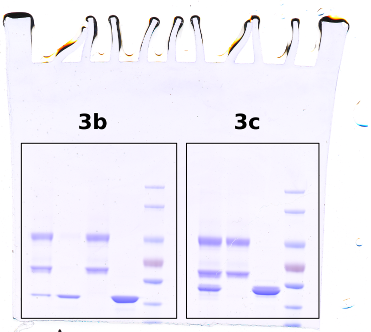

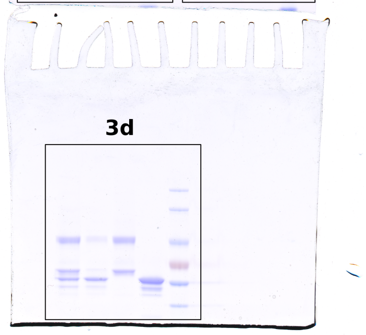

Suppl.Fig. 3c

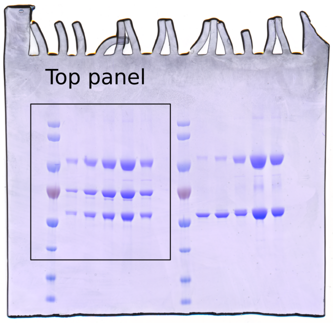

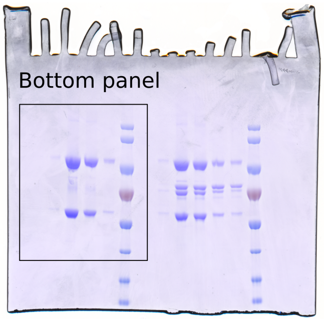

Suppl.Fig. 3b

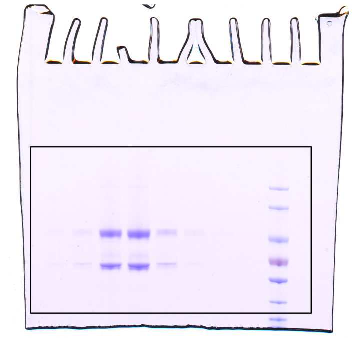

Top panel

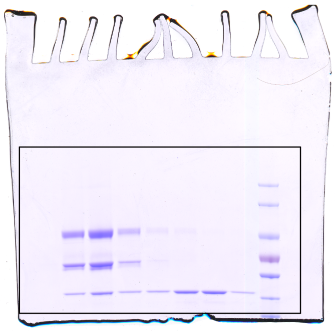

Second panel

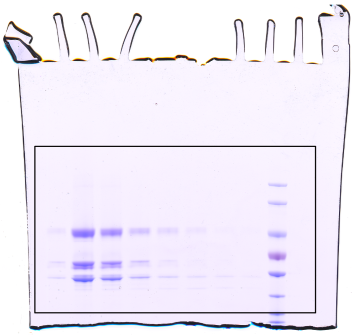

Third panel

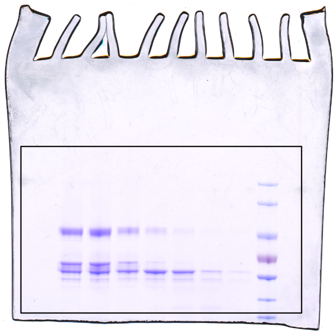

Bottom panel

Suppl.Fig. 3d

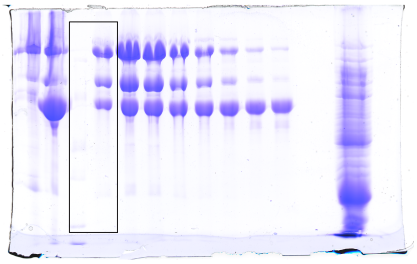

Raw gels for Fig.3 and Supplementary Fig. 3

5a (CBB)

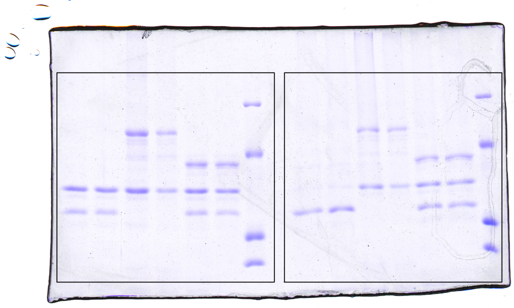

Input Precipitate

5a (^{32}P)

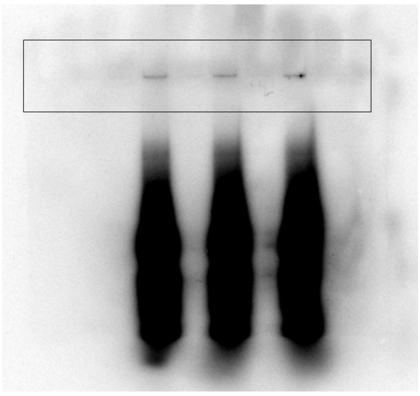

Input

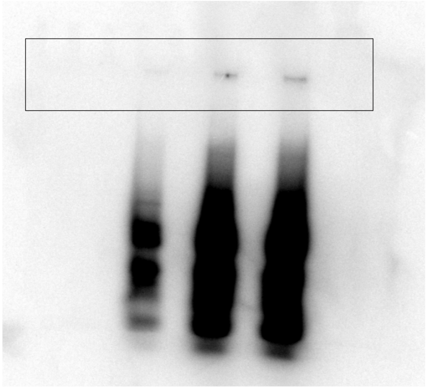

Precipitate

Suppl.Fig. 4b

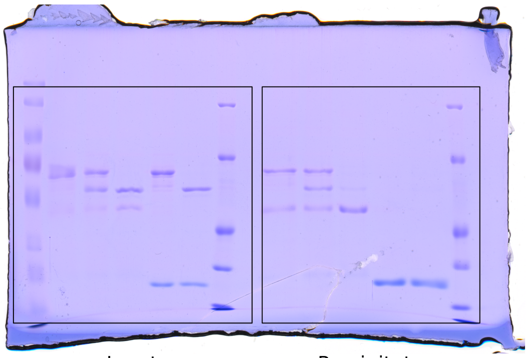

Input Precipitate

Raw gels for Fig.5 and Supplementary Fig. 4
