# Supplementary material for: Insights into the assembly and architecture of a Staufen-mediated mRNA decay (SMD)-competent mRNP
Source: Nat Commun. 2019 Nov 7;10:5054. doi: 10.1038/s41467-019-13080-x (PMC6838198; doi:10.1038/s41467-019-13080-x)
Supplement: Supplementary file 2 — Peer Review [file 41467_2019_13080_MOESM2_ESM.pdf]

Reviewers' comments:

Reviewer #1 (Remarks to the Author):

This paper presents careful in vitro work analyzing the interactions between Staufen 1 (Stau1) and UPF1. The data shows that in Staufen-mediated mRNA decay (SMD), UPF1 requires UPF2 for activation. These findings rectify previous publications from another group that concluded that UPF2 was not involved in SMD and that UPF2 and Stau1 were binding mutually exclusively to the CH domain of UPF1, which is important news for researchers interested in SMD.
Gowravaram and colleagues first showed that UPF1's ATPase activity is not stimulated but rather reduced in the presence of Stau1. The interaction of Stau1 with UPF1 is weaker than the interaction of UPF2 with UPF1 and does not withstand size exclusion chromatography. Furthermore, they showed that Stau1, apparently as a dimer and involving its dsRBD3, interacts with the MIF3G3 fragment of UPF2, suggesting that UPF1 is an adaptor between UPF1 and Stau1. The complex comprising UPF1, dimerized Stau1 dsRBD2-3 and UPF2 is stable during size exclusion chromatography. To collect further topological information about this complex, the authors performed X-linking mass spectrometry. From this information and based on available structural data, they present a structural model for the UPF1-UPF2-Stau1 ternary complex. Finally, the authors show that UPF2 is necessary for SMD of two SMD-targets in U2OS cells.
In summary, this is a sound study presenting well-controlled data and compelling conclusions. The topic is highly specialized and narrow and hence the paper will not be of interest for a very broad audience. However, for the researchers active in this field, the findings are important as they correct conclusions and derived models from previous papers.

Specific points:
The rationale for the experiment shown in Fig 5c was that ADAR1 antagonizes SMD and thus ADAR1 knockdown results in reduced mRNA levels of SMD-targets due to more active SMD. While that seems to be the case for CCNG1, ADAR1 seems not to antagonize SMD of RAD51 mRNA, since ADAR1 knockdown doesn't really change RAD51 mRNA abundance and hence the whole idea of rescuing RAD51 mRNA level with an additional knockdown of UPF1 or Stau1 becomes obsolete. Is there another ADAR1-sensitive SMD target that the authors could show to make their point?

Minor points:
p. 2: What is the evidence for the statement that SMD relies on efficient translation termination? Please clarify.
p. 2, Ref. 11: I recommend citing here a more recent NMD review, e.g. Kurosaki et al. Nat Rev Mol Cell Biol 2019, or Karousis and Muhlemann, CSH Perspect Biol 2018.
p. 3: The first sentence of the second paragraph should read "…not clear how the activity is stimulated in the context of SMD" and it needs a reference.

Reviewer #2 (Remarks to the Author):

Upf1 is an ATP-dependent RNA helicase that functions in a variety of eukaryotic mRNA decay pathways, such as nonsense-mediated decay (NMD). Its recruitment to transcripts is highly regulated, often triggered by recognition of cis-elements, such as premature termination codons during NMD or dsRNA found in the 3' UTR of transcripts during Staufen mediated decay (SMD). A major question in the field is how Upf1 is targeted and activated on specific transcripts. Prior work by Chakrabarti has elaborated the structure of Upf1 and how it is activated by UPf2 during NMD. The CH domain of Upf1 causes its helicase core to clamp on RNA with high-affinity, inhibiting helicase and ATPase function. Upf2 alleviates autoinhibition by binding the CH domain, remodeling its conformation so that it no longer interacts with the helicase core, which is free to promote ATP-dependent RNA unwinding and subsequent degradation by its interactions with nucleases.

Several observations from the Maquat lab led them to propose that Stau1 acts as a molecular and functional mimic of Upf2. Knockdown of Upf2 does not affect SMD (Kim et al, Cell 2005); Stau1 and Upf2 interact with a common domain of Upf1 (the CH domain) (Gong et al, G&D, 2009); there is an apparent competition between SMD and NMD in cells as ablation of Stau1 or Upf2 increases NMD or SMD respectively (Gong et al, G&D, 2009); and that Stau1 and Upf1 do not simultaneously coIP with Upf2 (Gong et al, G&D, 2009). However, it was never shown that Stau1 could directly stimulate Upf1, like Upf2, which is a central prediction of the mimicry model.

In the present study, Gowravaram et al provide data that suggest Stau1 and Upf2 work together to promote SMD, contrary to the aforementioned mimicry model. First, they show for the first time that, unlike Upf2, Stau1 does not activate Upf1 ATPase activity in vitro. Second, they provide evidence that instead of binding in a mutually exclusive manner as predicted by the mimicry model, both Upf2 and Stau1 bind Upf1 simultaneously. This is supported by several independent observations: i- Upf1 makes weak interactions with Stau1 in vitro; ii-Upf2 enhances the interaction of Stau1 with Upf1 as evidenced by GST-pull-down analyses and co-IP analysis in cells; iii-size exclusion chromatography analyses reveals Upf1 does not copurify with Stau1. In contrast, Upf2 allows detection of a stable heterotrimeric complex with Stau1 and UPf1 by SEC; iv-cross-linking MS analysis in conjunction with molecular modeling indicates Upf1 is the core of a Stau1 and Upf2 complex, where each protein occupies separate surfaces on Upf1 without molecular clashes. In support, the Upf2-Stau1 complex stimulates ATPase activity of UPf1 and knockdown of Upf2 or Stau1 increase the stability of endogenous SMD targets in cells. These data suggest Upf2 is a key player in SMD, just like in NMD. The model that emerges from these studies is that cooperative assembly of Stau1-Upf2-Upf1 on 3' UTR of mRNA allows targeting and activation of Upf1 on transcripts containing Staufen binding sites.

Overall the quality of the data is high, and the experiments are carefully executed. The observation that Upf2 helps Stau1 engage Upf1 is intriguing as is the functional dependence of Upf2 on SMD reported in Fig 5. While the findings are at variance with prior studies, they have the potential to clarify the mechanisms of how Upf1 is activated and targeted to transcripts in mRNA decay pathways outside of its role in NMD. The work could be suitable for publication in Nature Communications after the following points are addressed.

Major:

1- The effect of Upf2 on SMD was shown to be dispensable by Kim et al while the present work suggests it plays a pivotal role. Are there differences in the experimental design of Kim et al that could explain variance with data in Fig 5, which shows ablation of Upf2 stabilizes endogenous SMD substrates in cells? For example, could sensitizing cells to SMD by depleting ADAR contribute to this difference in Upf2 dependence? This discrepancy and potential differences in design between Kim et al and the present work should be acknowledged in the discussion.

2-Do published genome-wide studies support the notion that Upf2 and Stau1 work together to promote decay of a common set (SMD) targets? Stau1 binding sites have been identified in 3' UTR using RIPit and related approaches in mammalian cells and in drosophila embryos (Laver et al, NAR 2013; Ricci et al, NSMB 2014). Are target transcripts containing Stau1 binding sites statistically enriched in Upf2 sensitive transcripts? It may be possible to analyze existing data sets to address this question.

3-Do the authors have a plausible explanation for why Stau1 was not detected in Upf2 immunoprecipitates by Gong et al but robustly detected in their work?

4-Overall the biochemical data with reconstituted proteins is compelling, but several of the experiments are conducted with an entire region of Stau1 deleted. For example, replacing the tubulin binding domain (TBD) by the native dimerization domain of Stau1 or dimerization domains from

unrelated proteins(of HIP or NF-kB p50) results in a stable purification dsRMD 2-3-4 of Stau1 with Upf 1 and Upf2. The authors take advantage of this result for design and interpretation of experiments reported in Fig 3 and 4; however, is it possible that removal of the TBD results in a complex that is not biologically relevant? Can the authors justify the removal of the TBD?

5-The authors show that Stau1 forms direct interactions with Upf1, but that Upf2 augments formation of stable Stau1-Upf1 complex. Given this result, it is puzzling that Stau1-Upf2 does not stimulate Upf1 ATPase activity to a greater degree, as presumably, the Stau1-Upf2 module has a higher affinity for Upf1 than either cofactor alone. Is it possible that ATPase activities were performed under conditions where differences in binding affinity between Upf2 and Stau1-Upf2 for Upf1 are masked (i.e., Upf1 cofactors are present at saturating concentrations)? Does Stau1 decrease the concentration of Upf2 required for maximal activation of Upf1 ATPase activity?

Minor:

6-In Figure 1, why are different constructs of Upf2 employed in pull-down and ATPase assays (panel A versus panel D)?

7-The authors may wish to comment on why there no observed cross-links between Upf2 to the CH domain of Upf1 (despite established interactions born out by numerous biochemical, functional and structural studies).

8-Figure S3, a table comparing molecular weight predicted by primary sequence with molecular weight determined by SEC-MALS would be helpful.

Reviewer #3 (Remarks to the Author):

The authors report the characterization of in vitro interactions between UPF2, UPF2 and STAU1, leading to the unexpected conclusion that UPF1 is recruited and activated via a UPF2-STAU1 complex. The in vitro binding data presented support these conclusions. SMD is a significant mRNA turnover pathway and understanding of the key complex is important. However, some points should be addressed before I could recommend acceptance.

Specific comments:
1: It was previously reported that the STAU1- and UPF2-binding sites within UPF1 overlap, and evidence was presented for competition in vivo, leading to the conclusion that STAU1 and UPF2 binding to UPF1 are mutually exclusive (Gong et al. 2009). Moreover, STAU1 was reported to directly recruit UPF1 (Gong et al. 2013). These findings are alluded to in the Introduction but it would be very useful to explicitly discuss the basis for the differences in the conclusions.

2. The evidence for a requirement for UPF2 in SMD is based on suppression of the effects of ADAR1 depletion. An assay based on one or more characterized SMD substrate(s) would greatly strengthen the MS.

Minor points:

3: Figs. 1C and 1D: Differences reported by the authors are visible, but the data would be more compelling with quantitation.

4. p8: The results obtained by “fusing another dimerization domain” and “an obligate dimer of Stau1” should probably be better described or omitted.

5. Fig. 4a: It was not clear what is gained by duplicating the protein-protein interactions for the "second" STAU1 monomer. This distinction cannot be determined from the data and trans interactions with the two monomers are presumably largely mutually exclusive for each site, in contrast to what is apparently indicated.

Re: NCOMMS-19-10810-T

Insights from biochemical reconstitution into the assembly and architecture of a Staufen mediated mRNA decay (SMD)-competent mRNP

We would like to thank the reviewers for their constructive criticism and comments on our manuscript. All changes in the main text and figure legends have been highlighted in red, while additions or modifications to the supplementary information are mentioned here. We address the reviewers' comments in detail below:

Reviewer 1

Specific points:

- *The rationale for the experiment shown in Fig 5c was that ADAR1 antagonizes SMD and thus ADAR1 knockdown results in reduced mRNA levels of SMD-targets due to more active SMD. While that seems to be the case for CCNG1, ADAR1 seems not to antagonize SMD of RAD51 mRNA, since ADAR1 knockdown doesn't really change RAD51 mRNA abundance and hence the whole idea of rescuing RAD51 mRNA level with an additional knockdown of UPF1 or Stau1 becomes obsolete. Is there another ADAR1-sensitive SMD target that the authors could show to make their point?*

We have now investigated two additional transcripts, *MDM2* and *XIAP*, that were shown to be targets of ADAR1 (Yang et al., 2017, ref. 37 in the main text) and were additionally reported to be direct targets of Stau1 by Sugimoto, Ule and co-workers (Sugimoto et al., 2015, ref. 5 in the main text, please also refer to the response to Reviewer 3's comment below). Both *MDM2* and *XIAP* show a reduction in mRNA levels upon knockdown of ADAR1. Consistent with the targets being regulated by SMD, transcript levels are restored in cells where ADAR1 is knocked down in combination with either UPF2 or Stau1. These data are presented in the new Fig. 5.

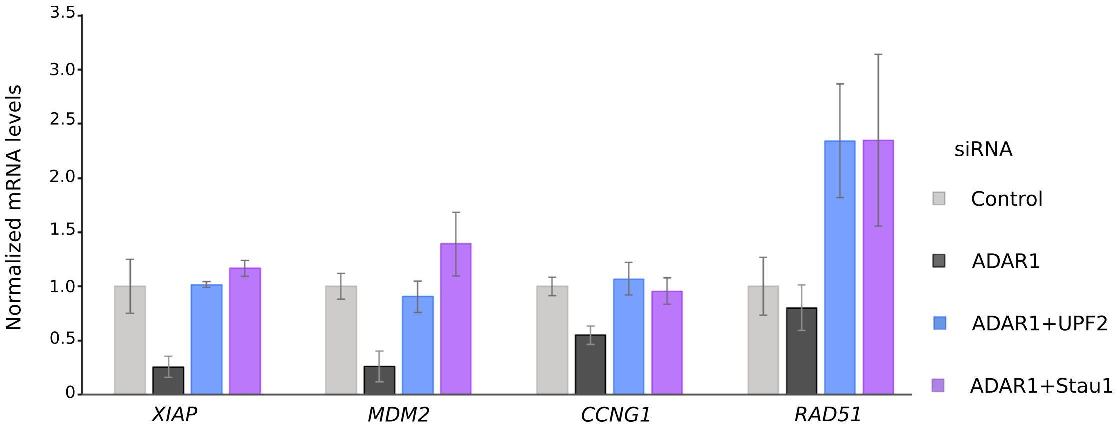

Minor points:

- *p. 2: What is the evidence for the statement that SMD relies on efficient translation termination? Please clarify.*

Kim, Maquat and co-workers observed that the presence of a termination codon upstream of the Staufen-binding site (SBS) is essential for mediating SMD. Moving the termination codon downstream of the SBS was shown to abrogate decay (Figure S2 of ref. 10 in the main text, Kim et al., 2005). Furthermore, Gong, Maquat and co-workers demonstrated that SMD is blocked upon treatment of cells with the translation elongation inhibitor, cycloheximide (Figure S3 of ref. 11 in the main text, Gong et al., 2009). Taken together, these observations suggest that SMD is dependent upon efficient translation. However, given that the observations of Kim, Maquat and co-workers (described above) could be simply due to removal of the bound Stau1 by the translating ribosome (a possibility alluded to by the authors in the paper), it is unclear if translation termination in particular has a specific role in SMD. Bearing this in mind, we have modified the statement in p.2 to "… relies not only on binding of Staufen to RNA but also on efficient translation and …" and have cited the work by Gong, Maquat and co-workers.

- *p. 2, Ref. 11: I recommend citing here a more recent NMD review, e.g. Kurosaki et al. Nat Rev Mol Cell Biol 2019, or Karousis and Muhlemann, CSH Perspect Biol 2018.*

We have replaced the previously cited NMD review with a more recent review from E.D. Karousis and O. Mühlemann (Karousis and Mühlemann, *Cold Spring Harb Perspect Biol*. 2019). This is ref. 12 of the modified manuscript.

- *p. 3: The first sentence of the second paragraph should read "...not clear how the activity is stimulated in the context of SMD" and it needs a reference.*

We have fixed this grammatical error in pg. 3 and have added a reference for the necessity of UPF1 helicase activity in SMD (Park et al., 2013, ref. 19 in the main text).

Reviewer 2

Major points:

1. The effect of Upf2 on SMD was shown to be dispensable by Kim et al while the present work suggests it plays a pivotal role. Are there differences in the experimental design of Kim et al that could explain variance with data in Fig 5, which shows ablation of Upf2 stabilizes endogenous SMD substrates in cells? For example, could sensitizing cells to SMD by depleting ADAR contribute to this difference in Upf2 dependence? This discrepancy and potential differences in design between Kim et al and the present work should be acknowledged in the discussion.

The experimental setup that we employed to test for the effect of UPF2 in SMD differs significantly from that used by Kim and co-workers in their study (Kim et al. 2005, ref. 10 in the main text). Whereas we monitored levels of specific cellular target mRNAs upon siRNA knockdown of UPF2 or Stau1 (together with ADAR1), Kim and co-workers investigated the effect of siRNA knockdown of UPF2 on a reporter mRNA to which Stau1 or UPF1 was tethered. Tethering of a protein on an mRNA significantly increases its local concentration as well as the residence-time on the RNA, and can therefore bypass the requirement for additional factors involved in the pathway. We conjecture that tethering of Stau1 or UPF1 to the mRNA circumvents the involvement of UPF2, whose main role in SMD is to recruit and activate UPF1. In the experimental setup employed by Kim and co-workers, the weak binding of UPF1 to Stau1 tethered to an mRNA reporter might be sufficient to recruit it and subsequently lead to degradation of the reporter. Similarly, tethering of UPF1 to the mRNA might overcome its need for activation by UPF2 and enable the helicase to use its basal catalytic activity to remodel the mRNP and facilitate degradation. The effect of knockdown of UPF2 on endogenous Stau1 targets was not investigated by Kim and co-workers.

Surprisingly, previously identified SMD targets such as *ARF1* or *SOWHAC* did not respond to downregulation or overexpression of Stau1, UPF1 or UPF2 in our hands. In this respect, our observation is similar to that reported by Ricci and co-workers (Ricci et al., 2014, ref. 4 in the main text). Therefore, we adapted the system reported by Sakurai, Nishikura and co-workers (Sakurai et al., 2017, ref. 36 in the main text) where we found that downregulating ADAR1 expression sensitizes certain mRNA transcripts (*CCNG1* and *RAD51*) to SMD. We have now investigated two additional mRNA transcripts (*XIAP* and *MDM2*), which are direct targets of Stau1 (please refer to the responses to comments from Reviewers 1 and 3). Both of these targets are also sensitive to knockdown of ADAR1 and show an upregulation upon combinatorial knockdown of ADAR1 and UPF2 or Stau1. It is possible that SMD typically occurs at low levels in the cells and that knockdown of the dsRNA-binding protein ADAR1 sensitizes many more mRNA transcripts containing dsRNA stretches in their 3'-UTR to SMD.

We have modified our discussion to reflect these differences in experimental setups and provide an explanation for why the involvement of UPF2 in SMD was not clear from previous studies.

2. Do published genome-wide studies support the notion that Upf2 and Stau1 work together to promote decay of a common set (SMD) targets? Stau1 binding sites have been identified in 3' UTR using RIPit and related approaches in mammalian cells and in drosophila embryos (Laver et al, NAR 2013; Ricci et al, NSMB 2014). Are target transcripts containing Stau1 binding sites statistically enriched in Upf2 sensitive transcripts? It may be possible to analyze existing data sets to address this question.

We analyzed two RNA-seq datasets from the ENCODE (Encyclopedia of DNA Elements) project (The ENCODE Project Consortium, 2012, Nature, 489, 57-74), where UPF2 and Stau1 had been knocked down in HepG2 cells using shRNA. Our goal was to observe the trend of known Stau1 targets (identified by Ricci and co-workers and Sugimoto and co-workers) in these cells. Interestingly, levels of common Stau1 targets (such as *ARF1*, *SOWHAC*, *XIAP*, *MDM2*) were lower in the Stau1 knockdown cells, in comparison to cells treated with the control shRNA. This unexpected trend corroborates our view that SMD typically occurs at very low levels in the cells and is probably induced under special circumstances (such as apoptosis or downregulation of ADAR1, as suggested by Sakurai and co-workers, ref. 36 in the main text). Staufen target mRNA levels were mostly unchanged or slightly reduced in the UPF2 knockdown cells but given the trend of their behavior in Stau1 knockdown cells, we conclude that this is not reflective of the role of UPF2 in SMD.

3. Do the authors have a plausible explanation for why Stau1 was not detected in Upf2 immunoprecipitates by Gong et al but robustly detected in their work?

In the study reported by Gong and co-workers (Gong et al. 2009, ref. 11 in the main text), the authors carried out co-immunoprecipitation assays in Cos cells with exogenously expressed, epitope tagged- UPF2, Stau1 and UPF1 constructs (Figure 2 of ref. 11 in the main text). The authors did not observe HA-Stau1 being co-precipitated with T7-UPF2 in their assays. Western Blot analyses to compare the levels of exogenous, tagged proteins with the corresponding endogenous, untagged proteins shows that the tagged proteins, particularly HA-Stau1, are expressed at significantly lower levels than their endogenous counterparts (Figure S1 of ref. 11 in the main text). We speculate that the reason that Gong and co-workers did not co-precipitate HA-Stau1 with T7-UPF2 is because most of the T7-UPF2 protein was bound to the endogenous Stau1, which was not detected by immunoblotting against the HA-tag. However, it should be mentioned that HA-Stau1 was co-precipitated with Myc-UPF1, albeit at very low levels. Although UPF1 was detected in immunoprecipitates of HA-Stau1, the presence of UPF2 in these samples was not checked for. In contrast to the study discussed above, our co-immunoprecipitation assays (Supplementary Fig. 1d) were carried out with tagged proteins over-expressed in HEK-293 cells, reducing the possibility of competition from endogenous factors. We are confident of the Stau1-UPF2 interaction that we report as this interaction could be recapitulated using purified proteins *in vitro*, free of any other cellular factors.

4. Overall the biochemical data with reconstituted proteins is compelling, but several of the experiments are conducted with an entire region of Stau1 deleted. For example, replacing the tubulin binding domain (TBD) by the native dimerization domain of

Stau1 or dimerization domains from unrelated proteins (of HIP or NF-kB p50) results in a stable purification dsRMD 2-3-4 of Stau1 with Upf1 and Upf2. The authors take advantage of this result for design and interpretation of experiments reported in Fig 3 and 4; however, is it possible that removal of the TBD results in a complex that is not biologically relevant? Can the authors justify the removal of the TBD?

We removed the entire TBD and fused the native or heterologous dimerization domains directly to the N-terminal dsRBDs to reinforce the argument that while the N-terminal dsRBDs comprise the interaction platform for UPF1 and UPF2 on Stau1, they are not sufficient to enable reconstitution of a stable ternary complex. Dimerization of Stau1 is essential as it strengthens the interaction of Stau1 with UPF1 and UPF2, explaining previous observations by Gleghorn and colleagues where Stau1 dimerization was found to be essential for SMD (Gleghorn et al. 2013, ref. 27 in the main text).

Nevertheless, we appreciate the reviewer's concern regarding removal of the TBD and therefore have reconstituted two additional ternary complexes to address this point: 1) UPF1-UPF2s-Stau1dsRBD2-3-4-TBD-DD, to illustrate that the presence of the TBD does not affect complex formation and 2) UPF1-UPF2 (MIF4G1-2-3-U1BD)-Stau1 full-length, to demonstrate the formation of a biologically relevant complex. These data have been added to the new Supplementary Fig. 3.

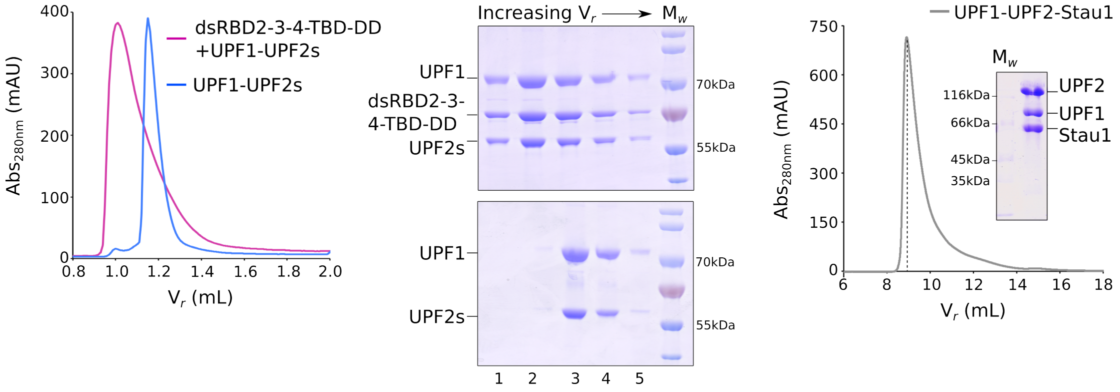

Furthermore, cross-linking mass-spectrometry analysis with the large UPF1-UPF2-Stau1 full-length complex (data not included in the manuscript) showed that many of the cross-links detected for this complex are also found within the minimal ternary complex used for the analysis in Fig. 4, suggesting that the topology of the minimal ternary complex is reminiscent of that consisting of the full-length proteins.

5. The authors show that Stau1 forms direct interactions with Upf1, but that Upf2 augments formation of stable Stau1-Upf1 complex. Given this result, it is puzzling that Stau1-Upf2 does not stimulate Upf1 ATPase activity to a greater degree, as presumably, the Stau1-Upf2 module has a higher affinity for Upf1 than either cofactor alone. Is it possible that ATPase activities were performed under conditions where differences in binding affinity between Upf2 and Stau1-Upf2 for Upf1 are masked (i.e., Upf1 cofactors are present at saturating concentrations)? Does Stau1 decrease the concentration of Upf2 required for maximal activation of Upf1 ATPase activity?

The ATPase assays reported in Fig. 5 and Supplementary Fig. 4 were performed with a mixture of Stau1, UPF1 and UPF2 proteins, where Stau1 and UPF2 were added in a 1.25-fold molar excess of UPF1. However, we also performed the ATPase assays with reconstituted complexes of Stau1, UPF1 and UPF2, where the proteins are present in equi-molar amounts (considering Stau1 as a dimer). We did not observe any differences in the trend of ATPase activities between UPF1-UPF2 and the UPF1-UPF2-Stau1 complexes in these two scenarios. We hypothesize that although UPF2 augments the binding of Stau1 to UPF1, the binding of UPF2 to UPF1, and as a resultant the extent of UPF1 activation, remains unaffected in the presence of Stau1. To test this hypothesis, we performed GST-pulldown assays where we compared the binding affinity of UPF2 to UPF1 in the absence and presence of Stau1. We found that the amount of UPF2 protein co-precipitated with UPF1 remains unchanged in the presence of Stau1. This data has been added to the new Supplementary Fig. 4.

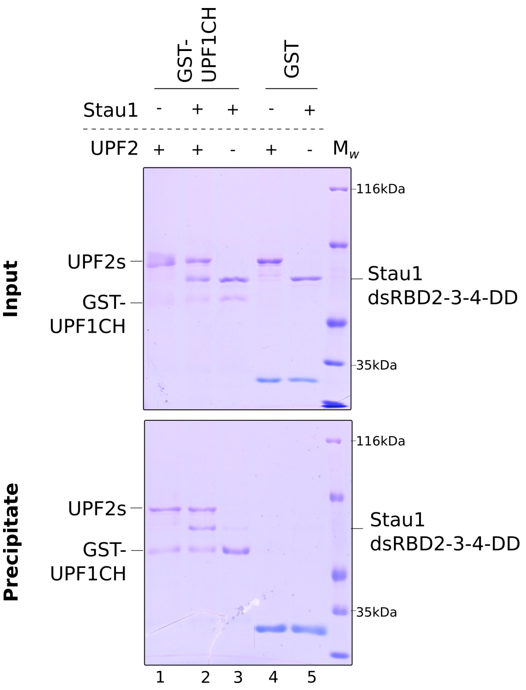

Minor points:

6. In Figure 1, why are different constructs of Upf2 employed in pull-down and ATPase assays (panel A versus panel D)?

In the ATPase assay in Fig. 1b, we used a truncation construct of UPF2 (UPF2s) that contains the UPF1 binding domain. This UPF2 construct was previously shown to robustly activate UPF1 (Chakrabarti et al., 2011, ref. 17 in main text). However, the problem with using this construct in the GST-pulldown assay in Fig. 1d is that UPF2s migrates at the same position as the Stau1 protein on a Tris-glycine-SDS gel (molecular weights of 54 kDa and 63 kDa for UPF2s and Stau1, respectively, also compare lanes 1 and 2 of Fig. 1c). Therefore, in order to clearly resolve all the proteins by SDS-PAGE, we resorted to using a longer construct of UPF2 that contains the MIF4G 1 and 2 domains in addition to the domains present in UPF2s.

7. The authors may wish to comment on why there are no observed cross-links between Upf2 to the CH domain of Upf1 (despite established interactions born out by numerous biochemical, functional and structural studies).

The complete absence of cross-links between the CH domain of UPF1 and the C-terminus of UPF2 in the cross-linking mass-spectrometric analysis of the ternary complex is indeed surprising. We assumed that this is either because the lysines within the CH domain of UPF1 are not favourably positioned to cross-link to the UPF2 C-terminus or because binding of Stau1 to the UPF1 CH domain, in addition to UPF2, restricts the accessibility of the chemical cross-linker to the lysine residues

present in the UPF1 CH domain. To distinguish between these two possibilities we have carried out cross-linking mass spectrometric analysis of the UPF1-UPF2s complex. In contrast to the ternary complex, we do find a few cross-links between the UPF1 CH domain and the UPF2 C-terminus. However, only 4 of the 11 lysines in the CH domain engage in cross-links with UPF2, suggesting that most of the lysines are not in a favourable position to carry out the cross-linking chemistry. As with the ternary complex, most of the UPF1-UPF2 cross-links involve the helicase core of UPF1 and the MIF4G3 domain of UPF2. The cross-linking data have been appended to the existing Supplementary Table 1.

8. Figure S3, a table comparing molecular weight predicted by primary sequence with molecular weight determined by SEC-MALS would be helpful.

We have updated Supplementary Fig. 3 to include a table with the theoretical molecular weights for both monomeric and dimeric forms of all Stau1 proteins (as calculated using the Expasy Protparam online tool) and the experimental molecular weights determined by SEC-MALS. Since these values are now presented in the figure, we have removed the theoretical molecular weights from the corresponding figure legend.

Reviewer 3

Major points:

1: It was previously reported that the STAU1- and UPF2-binding sites within UPF1 overlap, and evidence was presented for competition in vivo, leading to the conclusion that STAU1 and UPF2 binding to UPF1 are mutually exclusive (Gong et al. 2009). Moreover, STAU1 was reported to directly recruit UPF1 (Gong et al. 2013). These findings are alluded to in the Introduction but it would be very useful to explicitly discuss the basis for the differences in the conclusions.

We believe that the difference in the conclusions between the studies reported by Kim, Gong and co-workers and our study regarding the involvement and role of UPF2 in SMD stems from different experimental setups (please refer to the response to Reviewer 2's comments above). We have discussed these differences in experimental setups in the discussion section and explain how our observations reconcile with the model suggesting that NMD and SMD are competitive pathways *in vivo*.

2. The evidence for a requirement for UPF2 in SMD is based on suppression of the effects of ADAR1 depletion. An assay based on one or more characterized SMD substrate(s) would greatly strengthen the MS.

Unfortunately, the commonly known SMD targets such as *ARF1* failed to show any effect upon Stau1 knockdown or upregulation in our hands. This is consistent with data from Melissa J. Moore's laboratory (Ricci et al., 2014, ref. 4 in the main text). Furthermore, luciferase reporters containing the Staufen binding site (SBS) from the *ARF1* 3'-UTR showed no effect upon Stau1 knockdown (data not included in the manuscript). This led to our conclusions that SMD occurs at very low levels in the cell and has to be triggered through a specific mechanism. Keeping this in mind, we adapted the setup reported by Sakurai and co-workers (Sakurai et al., 2017, ref. 36 in the main text) where knockdown of ADAR1 sensitizes mRNA transcripts to SMD. Using this system, we had initially tested the effects of ADAR1/UPF2 knockdown on the levels of two mRNA transcripts (*CCNG1* and *RAD51*), which are primary targets of ADAR1. We have now investigated the levels of two additional mRNA transcripts, *XIAP* and *MDM2*. All four target mRNA transcripts investigated were reported to be direct targets of Stau1 by Sugimoto, Ule and co-workers (Sugimoto et al., 2015, ref. 5 in the main text). *XIAP* and *RAD51* were also identified as Stau1 targets by Ricci and co-workers. Both *XIAP* and *MDM2* are known anti-apoptotic factors and also respond to knockdown of ADAR1 and, therefore, are suitable targets to investigate in our experimental system (please refer to the response to the comments from Reviewers 1 and 2 above, particularly the figure added in response to Reviewer 1's comment). These new data have been added to Fig. 5 of the revised manuscript.

Minor points:

3: Figs. 1C and 1D: Differences reported by the authors are visible, but the data would be more compelling with quantitation.

We have quantified the intensity of the Stau1 and UPF2 bands of Fig. 1c (lower panel) and the intensity of the Stau1 bands in Fig. 1d (lower panel) and have added the results of the quantitation to the respective figure legends and main text.

4. p8: The results obtained by "fusing another dimerization domain" and "an obligate dimer of Stau1" should probably be better described or omitted.

Since the experiments in Fig. 3 were carried out with Stau1 constructs fused to a heterologous dimerization domain different from that used in Fig. 2, we have deleted the statement "verified by fusing with another dimerization domain" from the main text. For the interaction of UPF1 with Stau1, we have elaborated on what is meant by "an obligate dimer" (a strong Stau1 dimer mediated by a heterologous dimerization domain such as that of p50 and Hip).

5. Fig. 4a: It was not clear what is gained by duplicating the protein-protein interactions for the "second" STAU1 monomer. This distinction cannot be determined from the data and trans interactions with the two monomers are presumably largely mutually exclusive for each site, in contrast to what is apparently indicated.

We concur with the reviewer in that showing the interactions with the second Stau1 monomer might be confusing for the reader. Our original intention was to indicate that the interactions between UPF1/UPF2 and Stau1 might take place with either monomer (though not with both simultaneously). We have modified Fig. 4 to show inter-links with only one monomer of Stau1; the second monomer has been included in the figure to represent inter-links between the Stau1 monomers. Accordingly, the figure legend has been modified to read, "Inter-links are only shown with one Stau1 monomer, as the two molecules within the dimer cannot be distinguished in our study".

REVIEWERS' COMMENTS:

Reviewer #1 (Remarks to the Author):

My points have all been satisfactory addressed in the revised version and I recommend acceptance of the manuscript.
I also noticed that several of the points raised by Reviewers 2 and 3 were about requesting explanations from the authors why their results differ from previously published work. Given that the data in this manuscript is carefully controlled and compelling, I find that the authors should not be pushed to give reasons in their paper why the other work was not reproducible. It is sufficient to state the other work that claimed SMD was independent of UPF2 (which is a negative result and hence in a strict sense not conclusive).

Reviewer #2 (Remarks to the Author):

The revised manuscript answers my questions, and I think those of the other reviewers, and it is now suitable for publication in Nature Communications.

Reviewer #3 (Remarks to the Author):

The authors have generally responded well to the comments of the referees and I would support publication of the revised MS.

Figs. 1C and 1D: When I suggested that the data would be more compelling with quantitation, I had envisaged something more precise than “approximately 3-fold”. I assume this means that the authors do not have quantitative data for the gels. This is unfortunate, but I would not make it a condition of publication.
